# Multi-scale convolution with efficient channel attention improves benign–malignant classification of spinal tumors on T2-weighted fat-suppressed MRI

**DOI:** 10.3389/fonc.2026.1951483

**Published:** 2026-10-30

**Authors:** Zhiwei Ma, Xingyu Duan, Xiaorong Ma, Cuiping Yan, Xuewei Wang, Ningkui Niu

**Affiliations:** 1 Department of Orthopedics, The Third People’s Hospital of Yinchuan, Yinchuan, China; 2 Department of Orthopedics, General Hospital of Ningxia Medical University, Yinchuan, China; 3 Department of Anesthesiology, The Third People’s Hospital of Yinchuan, Yinchuan, China; 4 Department of Respiratory Medicine, The Third People’s Hospital of Yinchuan, Yinchuan, China; 5 Medical Records Department, General Hospital of Ningxia Medical University, Yinchuan, China; 6 Research Center for Prevention and Control of Bone and Joint Tuberculosis, General Hospital of Ningxia Medical University, Yinchuan, China; 7 Key Laboratory of Clinical Pathogenic Microorganisms, General Hospital of Ningxia Medical University, Yinchuan, China

**Keywords:** benign–malignant classification, deep learning, medical image analysis, spinal tumor, T2-weighted MR imaging

## Abstract

**Background:**

Accurate differentiation between benign and malignant spinal tumors is critical for treatment planning, yet manual interpretation of T2-weighted fat-suppressed MRI remains subjective, with substantial inter-observer variability. This study aimed to develop a deep learning model, the multi-scale deep residual network (MSDR-Net), for automated benign–malignant classification.

**Methods:**

A total of 500 T2-weighted fat-suppressed MR images of pathologically confirmed spinal tumors (389 benign, 111 malignant) were retrospectively collected from two institutions. Data were partitioned into training (70%), validation (20%), and independent test (10%) sets, and five-fold cross-validation was performed. Leave-one-center-out external validation was also performed (development: Institution A, n = 300; evaluation: Institution B, n = 200). The proposed MSDR-Net incorporates multi-branch residual blocks that combine parallel multi-scale convolutional paths with efficient channel attention modules and was benchmarked against six established architectures.

**Results:**

On the independent test set (N = 50; 12 malignant cases), MSDR-Net achieved 92.0% accuracy, 91.7% sensitivity (95% CI 61.5%–99.8%), 92.1% specificity, and an AUC of 0.921, the highest point estimates among evaluated architectures. The model was well calibrated (Brier score 0.072), with net benefit over treat-all strategies across thresholds of 0.10–0.60. Derived from a small test cohort, these estimates carry substantial uncertainty and await confirmation in larger, prospective cohorts. External-cohort accuracy was 86.5% (AUC 0.875). Ablation experiments showed that removing either component degraded performance, with a modest super-additive interaction; given their substantially smaller capacity, the single-path variants’ deficit cannot be attributed solely to removing multi-scale processing. The model has 55.4M parameters and 12.3-ms inference per image.

**Conclusion:**

MSDR-Net effectively captures multi-scale discriminative features of spinal tumors on T2-weighted fat-suppressed MRI, achieving high classification accuracy while maintaining computational efficiency. The model shows promise for clinical deployment as an auxiliary diagnostic tool for orthopedic surgeons and radiologists.

## 1 Introduction

Spinal tumors are clinically important lesions for which accurate benign–malignant differentiation directly determines treatment selection and prognostic assessment (1, 2). Benign lesions are typically managed conservatively or with local excision, whereas their malignant counterparts necessitate aggressive surgical resection combined with radiotherapy and/or chemotherapy. Consequently, misdiagnosis may result in either delayed intervention or overtreatment (3, 4). T2-weighted fat-suppressed MRI is routinely used in the diagnostic work-up of spinal tumors (5). Reported inter-observer agreement for benign–malignant characterization is moderate to substantial (6); in our own reader study, pairwise Cohen’s kappa values ranged from 0.71 to 0.76 (Section 3.8). However, manual interpretation remains highly subjective and heavily dependent on radiologists’ expertise, particularly in cases involving early-stage subtle lesions, atypical presentations, or overlapping imaging features (7, 8). Therefore, developing a highly accurate and reproducible automated decision-support system is a pressing clinical need.

In recent years, deep learning has demonstrated considerable potential in medical image analysis (9–11). In particular, deep neural networks have achieved strong performance in tumor detection and classification across diverse anatomical sites and imaging modalities, including brain tumors on CT and MRI (12–14), breast cancer (15), and lung cancer on CT (16, 17). Nevertheless, general-purpose models face considerable challenges when applied to spinal tumor classification. First, spinal T2-weighted fat-suppressed MR images exhibit complex anatomical structures and highly variable tumor morphology, making it difficult for standard convolutional neural networks to capture fine-grained local detail and global context simultaneously. Second, early-stage lesions are frequently small and low-contrast, making single-scale feature extraction prone to the loss of diagnostically critical information (18–20). To address these limitations, we propose a multi-scale deep residual network (MSDR-Net). The network combines parallel multi-scale convolutional pathways of different kernel sizes with an efficient channel attention mechanism, enabling precise extraction and selective enhancement of tumor-relevant features. This architecture improves detection sensitivity for early-stage lesions while enhancing classification robustness and maintaining computational efficiency, and it may serve as a decision-support tool that helps radiologists streamline clinical workflows.

The contributions of this study are threefold: (1) a multi-branch residual block with parallel multi-scale convolutional pathways tailored to the scale heterogeneity of spinal tumors on T2-weighted fat-suppressed MRI; (2) integration of dimensionality-preserving efficient channel attention to suppress background anatomical interference; and (3) a comprehensive evaluation framework encompassing five-fold cross-validation, paired statistical testing, calibration and decision-curve analysis, leave-one-center-out external validation, a blinded reader study, and deployment profiling.

## 2 Materials and methods

### 2.1 Data collection and ethics

This study employed a retrospective dual-center study design. Data were obtained from the General Hospital of Ningxia Medical University and The Third People’s Hospital of Yinchuan between January 2022 and November 2025. The inclusion criteria were as follows: (1) pathologically confirmed spinal tumor diagnosis (via biopsy or postoperative specimen); (2) availability of a single standard T2-weighted fat-suppressed MR image per patient, with other sequences such as T1-weighted and contrast-enhanced images excluded to ensure input consistency; and (3) clearly identifiable tumor lesions on imaging. The exclusion criteria included: (1) severe motion artifacts or poor image quality precluding reliable diagnostic assessment; (2) concomitant severe spinal deformity, trauma, or surgical history interfering with tumor evaluation; and (3) incomplete or missing pathological records.

#### 2.1.1 Image selection

All included images were sagittal T2-weighted fat-suppressed acquisitions, and a single standardized slice was used for every case. For each examination, one musculoskeletal radiologist with 12 years of experience selected the representative image according to a prespecified criterion—the slice showing the largest lesion cross-sectional area. This radiologist was not involved in ROI delineation, was blinded to the pathological diagnosis and all model outputs, and completed all image selection before any model development began. For multifocal tumors (n = 23), the lesion with the largest cross-sectional area on the selected slice was designated the index lesion, and only that lesion was analyzed.

A total of 500 pathologically confirmed spinal tumor cases were finally enrolled, comprising 389 benign cases (77.8%) and 111 malignant cases (22.2%). Benign tumors included osteochondromas (n = 98), hemangiomas (n = 175), giant cell tumors (n = 18), and other benign lesions (n = 98). Malignant tumors included metastatic tumors (n = 86), multiple myelomas (n = 17), and chordomas and chondrosarcomas (n = 8). Primary tumors comprised 414 cases (82.8%), including 389 benign lesions and 25 primary malignancies (17 multiple myelomas and 8 chordomas/chondrosarcomas); metastatic tumors accounted for 86 cases (17.2%, all malignant). The patient enrollment and dataset partitioning strategy is illustrated in **Figure 1**.

**Figure 1:**
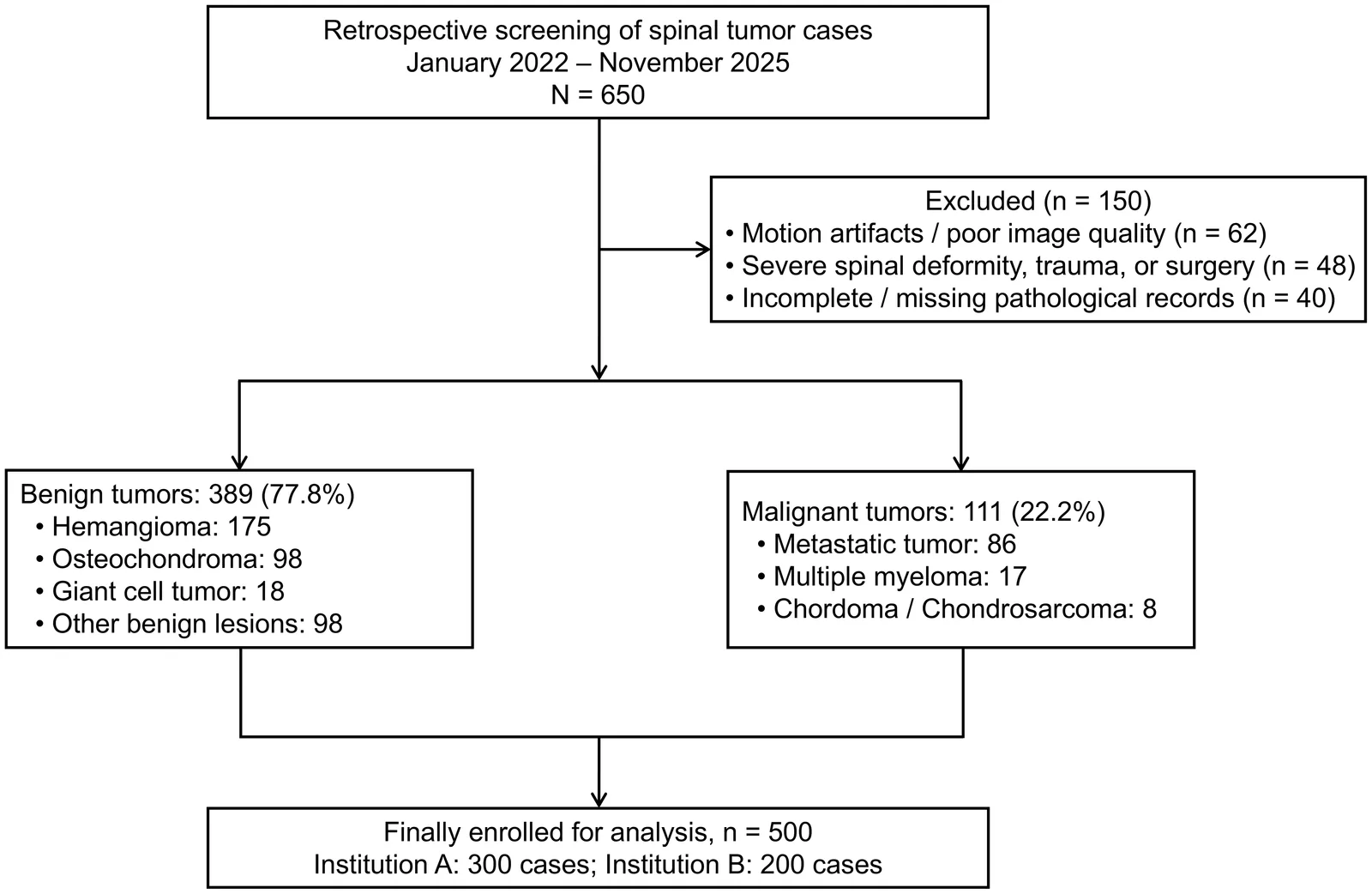
Patient enrollment and dataset partitioning flowchart.

#### 2.1.2 Dataset partitioning strategy

Stratified random sampling was employed to approximate the overall benign-to-malignant ratio in each subset. All partitioning, including the 7:2:1 split and five-fold cross-validation fold assignment, was conducted at the patient level, ensuring that each patient appeared in only one subset and one-fold.

- Training set: 350 cases (273 benign, 77 malignant), used for model parameter optimization;
- Validation set: 100 cases (78 benign, 22 malignant), used for hyperparameter tuning, early-stopping monitoring, and model selection;
- Independent test set: 50 cases (38 benign, 12 malignant), reserved exclusively for final performance evaluation, with no involvement in any training or hyperparameter adjustment procedures.

Furthermore, to assess model stability, five-fold stratified cross-validation was performed on the combined training and validation sets (n = 450), with each fold maintaining approximately identical class proportions (each fold containing approximately 70 benign and 20 malignant cases, differing by at most one case across folds).

This study was approved by the Ethics Committee of the General Hospital of Ningxia Medical University (approval number: KYLL-2026-0784); this approval covers both participating institutions under an inter-institutional data use agreement. Given the retrospective use of anonymized imaging data, the requirement for individual informed consent was waived in accordance with the Regulations on Ethical Review of Biomedical Research Involving Humans. All patient-identifying information was de-identified prior to analysis. Each patient contributed only one image, and dataset partitioning was strictly performed at the patient level (case level), eliminating data leakage risks.

### 2.2 Data preprocessing and annotation quality control

#### 2.2.1 DICOM conversion

All raw DICOM images were first rescaled using the rescale slope and intercept values stored in the DICOM header. Signal intensities were then clipped to the 0.5th–99.5th percentile range of each individual image volume to suppress extreme values, and the processed images were exported as lossless 16-bit PNG files to preserve the full grayscale dynamic range.

#### 2.2.2 Step 1: region of interest extraction

A semi-automated workflow was utilized to extract the spinal segment containing the lesion. The automated component is a fixed, rule-based cropping procedure: an initial crop of fixed size (160 × 160 pixels) is centered on the seed point placed by the radiologist at the involved vertebral level and is then expanded in 4-pixel steps in each of the four directions; expansion along each direction stops when the mean intensity gradient along the corresponding crop edge falls below a prespecified threshold (0.05 in normalized intensity units), indicating that the vertebral body boundary has been reached, or when the crop reaches 1.5× the initial size, whichever occurs first. The rule-based cropping script and all parameter values are available in the public code repository. This procedure contains no trainable parameters and was never fitted on any study image; it is therefore entirely independent of the dataset partitioning, and no information from the validation or test cohorts could enter either ROI extraction or classifier training. To validate the rule-based extraction, 50 cases were randomly sampled, and agreement against manual reference delineations yielded a mean Dice similarity coefficient of 0.88 ± 0.05.

Tumor boundaries were independently delineated by two chief physicians, each with over 10 years of experience in spinal imaging. Inter-reader delineation agreement was quantified using the pixel-level Dice similarity coefficient (DSC) and Intersection over Union (IoU), with the 95th-percentile Hausdorff distance (HD95) reported as a boundary-distance metric. Cases with an inter-reader DSC < 0.80 were adjudicated by a third senior expert with over 20 years of experience to determine the final labels. Across all 500 cases, mean inter-reader agreement was DSC 0.87 ± 0.06, IoU 0.78 ± 0.08, and HD95 3.4 mm (**Supplementary Table S4**). For cases with unclear boundaries (8%, n = 40), precise manual cropping was performed to ensure lesion integrity. Baseline characteristics showed no significant differences between manually cropped (n = 40) and automatically extracted (n = 460) cases in benign-to-malignant ratio (p = 0.72), age distribution (p = 0.68), pathological subtype composition (p = 0.55), lesion size (p = 0.63), or T2 signal heterogeneity (p = 0.58); nevertheless, these comparisons cannot fully exclude selection bias related to boundary visibility, which is acknowledged as a limitation. All ROIs were uniformly resized to 224 × 224 pixels using bilinear interpolation.

While semi-automated delineation was necessary in this study to isolate algorithm performance, real-world deployment will integrate an automated spine/vertebra detection module (e.g., YOLO or nnU-Net) for ROI initialization within PACS workflows, requiring only physician confirmation. Long-term transition to whole-image weakly supervised learning is planned to eliminate ROI dependency entirely.

#### 2.2.3 Step 2: image enhancement and normalization

First, image-wise Z-score normalization was applied to standardize pixel values to zero mean and unit variance (Equation 1):

(1) $$x'=\frac{x-\mu }{\sigma }$$

where *x* denotes the original pixel value, and *μ* and *σ* represent the mean and standard deviation of the pixel values of each image, respectively.

Subsequently, contrast limited adaptive histogram equalization (CLAHE) was applied with a tile size of 8 × 8 and a contrast clipping limit of 2.0 to enhance lesion edge details; Z-score normalization was always performed before CLAHE. Finally, single-channel grayscale images were replicated into three channels (simulating RGB format) to accommodate the input requirements of ImageNet-pretrained comparison models and to unify the input pipeline across all experiments. All preprocessing parameters (normalization statistics, CLAHE parameters, and resize interpolation methods) were serialized and stored to ensure full experimental reproducibility.

### 2.3 Network architecture design

This study proposes the Multi-Scale Deep Residual Network (MSDR-Net), featuring a five-level encoder architecture that accepts 224 × 224 × 3 input images and outputs binary classification probabilities via a multilayer perceptron (MLP) (**Figure 2**). Layer-by-layer specifications are provided in **Supplementary Table S5**; the forward propagation is fully defined by the formulations in Sections 2.3.1–2.3.3, and the implementation is available in the public code repository.

**Figure 2:**
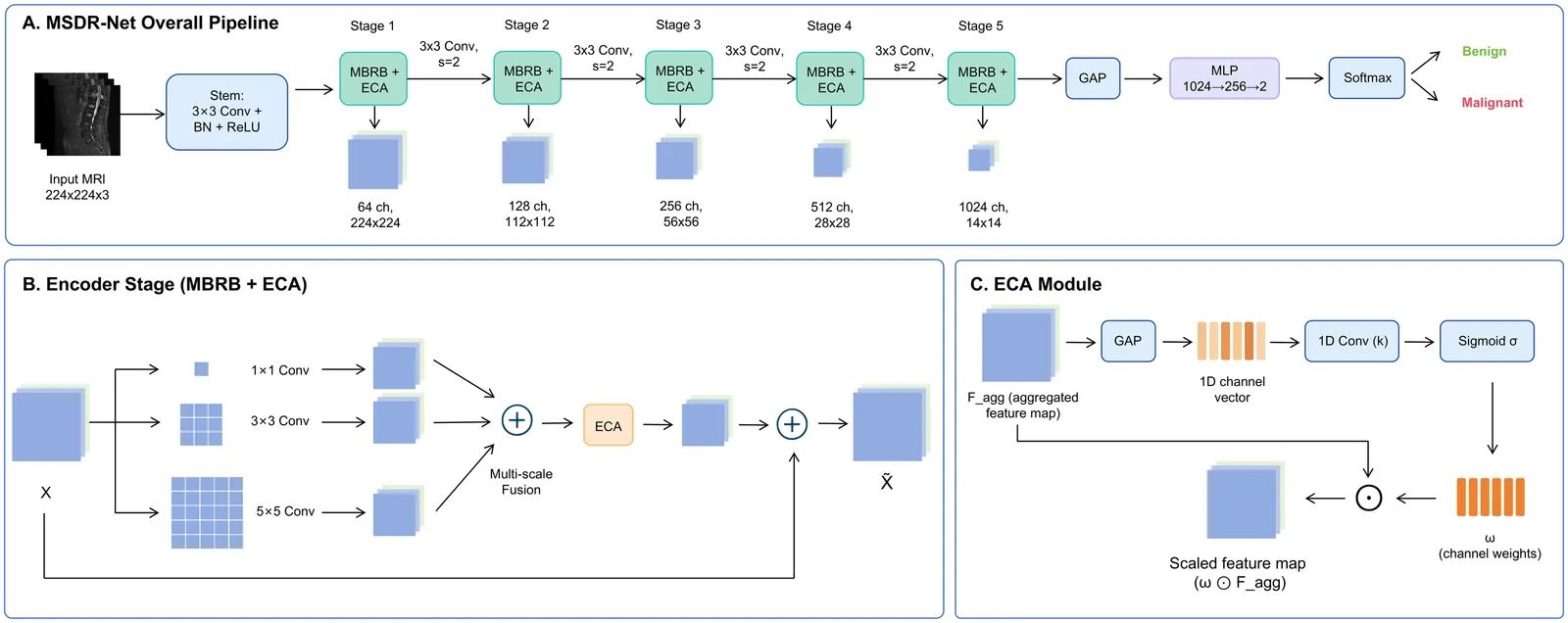
Architecture of MSDR-Net. **(A)** A stem (3 × 3 Conv + BN + ReLU), five encoder stages (MBRB + ECA) linked by stride-2 3 × 3 convolutions that halve the spatial size and double the channels (64–1024), then GAP and an MLP head (1024→256→2) with Softmax for benign/malignant classification. **(B)** Parallel 1 × 1, 3 × 3, and 5 × 5 branches fused by element-wise addition, followed by ECA and an identity residual connection. **(C)** GAP, an adaptive 1D convolution (kernel k), and sigmoid gating yield channel weights ω to rescale the feature map (k = 3, Stage 1; k = 5, Stages 2–5).

#### 2.3.1 Multi-branch residual block

Let $X\in R^{C\times H\times W}$ denote the feature map input to the current multi-branch residual module, where *C*, *H*, and *W* denote the number of input channels, the height, and the width of the feature map, respectively. The input feature *X* is simultaneously fed into three parallel convolutional pathways with distinct local receptive fields. To capture multi-scale lesion textures while maintaining parameter efficiency, the forward propagation of each branch is formulated as (Equation 2):

(2) $$F_{i}=W_{i}*X+b_{i},\;i\in \{1,2,3\}$$

where * denotes the standard convolution operation, and *W_i_* and *b_i_* represent the weight matrix and bias vector of the corresponding branch, respectively. The three pathways are:

- Path 1 (point-level features): a standard 1 × 1 convolution to capture fine local details and point-level feature responses;
- Path 2 (local textures): a standard 3 × 3 convolution to extract local texture patterns and edge information;
- Path 3 (regional context): a standard 5 × 5 convolution to capture regional morphological context and surrounding tissue relationships.

Subsequently, the spatial feature maps of these three scales are fused via multi-scale integration through an element-wise addition operation, yielding the aggregated feature map *F*_agg_ (Equation 3):

(3) $$F_{\text{agg}}=F_{1}⊕F_{2}⊕F_{3}$$

where ⊕ denotes element-wise addition.

#### 2.3.2 Efficient channel attention without dimensionality reduction

The aggregated feature *F*_agg_ is subsequently fed into an efficient channel attention (ECA) module (21) to capture cross-channel interactive information. First, global average pooling (GAP) is employed to map the three-dimensional spatial features into a one-dimensional channel descriptor $z\in R^{C}$, whose scalar value for the *c*-th channel is computed as (Equation 4):

(4) $$z_{c}=\frac{1}{H\times W}\overset{H}{\underset{i=1}{\sum }}\overset{W}{\underset{j=1}{\sum }}F_{\text{agg},\;c}(i,j)$$

To avoid the negative impact of dimensionality reduction in traditional attention mechanisms, the ECA mechanism directly computes the channel weights $\omega \in {\text{R}}^{C}$ without dimensionality reduction through a one-dimensional convolution with an adaptive kernel size *k* (Equation 5):

(5) $$\omega =\sigma (C1D_{k}(z))$$

where *σ* denotes the sigmoid activation function, and C1D*_k_* denotes a one-dimensional convolution of kernel size *k* whose parameters are shared across channels. The adaptive kernel size *k* is determined by a nonlinear mapping of the total number of channels *C* (Equation 6 and Equation 7):

(6) $$k=\psi (C)=|\frac{\log_{2}(C)}{\gamma }+\frac{b}{\gamma }{|}_{\text{odd}}$$

(7) $$|t{|}_{\text{odd}}=\{\begin{array}{c} \lfloor t\rfloor ,\lfloor t\rfloor \;isodd\\ \lfloor t\rfloor +1,\lfloor t\rfloor \;iseven \end{array}$$

where the constants *γ* and *b* are set to 2 and 1, respectively.

Finally, the computed channel weight vector *ω* is multiplied with the multi-scale spatial feature map via a channel-wise element-wise product (denoted ⊙) and fused with the original input *X* through an identity-mapping residual connection, yielding the final enhanced feature map $\tilde{X}$ (Equation 8):

(8) $$\tilde{X}=(\omega ⊙F_{agg})⊕X$$

#### 2.3.3 Encoder architecture and classification head

The network employs five progressive encoding stages (**Table 1**), each consisting of an MBRB followed by an ECA module. Between consecutive stages, spatial dimensions are halved using a 3 × 3 convolution with stride 2 and padding 1, while the number of channels is doubled (64, 128, 256, 512, and 1024 channels across Stages 1–5, respectively). After the final encoding stage, global average pooling aggregates the spatial features, and an MLP classification head outputs the binary (benign/malignant) class probabilities.

**Table 1:** Encoder architecture specifications of the proposed MSDR-Net.

Stage	Operation	Output channels	Spatial dimensions	Downsampling method
Input	—	3	224×224	—
Stem	3×3 Conv + BN + ReLU	64	224×224	—
Stage 1	MBRB + ECA	64	224×224	—
Stage 2	MBRB + ECA	128	112×112	3×3 Conv, stride=2, padding=1
Stage 3	MBRB + ECA	256	56×56	3×3 Conv, stride=2, padding=1
Stage 4	MBRB + ECA	512	28×28	3×3 Conv, stride=2, padding=1
Stage 5	MBRB + ECA	1024	14×14	3×3 Conv, stride=2, padding=1
Classifier head	Global average pooling + MLP (1024→256→2)	2	1×1	—

MBRB, multi-branch residual block; ECA, efficient channel attention; BN, batch normalization. Downsampling is performed by the 3×3 stride-2 convolution at the entry of Stages 2–5. Tensor dimensions are consistent with **Figure 2** and **Supplementary Table S5** throughout.

### 2.4 Loss function and class weighting

Weighted cross-entropy loss was employed to address class imbalance (Equation 9):

(9) $$L_{\text{WCE}}=-\frac{1}{N}\overset{N}{\underset{i=1}{\sum }}w_{y_{i}}\log p_{i,\;y_{i}}$$

where *N* denotes the number of training samples, *y_i_* the true class label of sample *i*, $p_{i,\;y_{i}}$ the predicted probability assigned to the true class, and $w_{y_{i}}$ the weight of the class to which sample *i* belongs. Class weights were calculated from the inverse class frequency in the training set (Equation 10):

(10) $$w_{c}=\frac{N}{K\cdot N_{c}}$$

where *N_c_* denotes the number of samples of class *c*, and *K* = 2 the number of classes. Substituting the training-set data ( $N_{\text{pos}}=77$, $N_{\text{neg}}=273$, N=350) yields $w_{\text{pos}}\approx 2.27$ and $w_{\text{neg}}\approx 0.64$. In cross-validation experiments, class weights were recomputed within the training portion of each fold according to that fold’s class frequencies.

### 2.5 Optimization and training schedule

#### 2.5.1 Data augmentation

Data augmentation was applied to the training data only and comprised random horizontal flipping (probability 0.5), random rotation within ±10°, random translation of up to 5% of the image dimensions, and random brightness/contrast perturbation within ±10%.

#### 2.5.2 Optimization

The AdamW optimizer was used (weight decay λ=0.01, $\beta_{1}=0.9$, $\beta_{2}=0.999$) with an initial learning rate of $10^{-4}$. A MultiStepLR schedule was adopted, multiplying the learning rate by a decay factor of 0.1 at epochs 80 and 140. Training proceeded for 200 epochs with a batch size of 16. Hyperparameters were selected from the search space summarized in **Supplementary Table S6**, with the configuration achieving the highest mean five-fold cross-validation AUC on the combined training/validation set carried forward (Section 2.6, stage 2).

#### 2.5.3 Initialization, repeated training, and reproducibility

MSDR-Net was randomly initialized using Kaiming initialization, as no ImageNet-pretrained weights are available for this custom architecture. All baseline models were fine-tuned from ImageNet-1K pretrained weights with the entire backbone fully unfrozen. Each architecture was trained three times independently (random seeds 2021, 2022, and 2023) with cuDNN deterministic mode enabled and benchmark mode disabled, and the run with the highest validation AUC was carried forward to test-set evaluation (**Supplementary Table S8**).

#### 2.5.4 Hardware environment and software

The model was implemented in the PyTorch 2.1.0 framework and trained on a single NVIDIA GeForce RTX 4080 GPU (16 GB VRAM). Automatic mixed precision (AMP) was employed to accelerate training and reduce VRAM consumption.

### 2.6 Model development and test-set locking protocol

The model development timeline proceeded as follows. (1) Test-set locking: the 50-case independent test set was locked before any modeling began and was never used for any design, tuning, or selection decision. (2) Configuration selection: candidate architecture configurations and the hyperparameter grid of **Supplementary Table S6** were compared by five-fold cross-validation on the combined 450-case training/validation set, and the configuration achieving the highest mean cross-validation AUC was adopted and frozen; cross-validation served a selection role only at this stage. (3) Final model training: the frozen configuration was trained on the 350-case training set, with early stopping and checkpointing monitored on the 100-case validation set (monitoring metric: validation AUC; patience: 20 epochs; checkpoint taken at the best validation AUC); three independent runs (random seeds 2021, 2022, and 2023) were performed, and the run with the highest validation AUC was carried forward to testing (**Supplementary Table S8**). (4) Stability assessment: only after the configuration and the checkpointing rule had been frozen was five-fold cross-validation re-run—once per fold with a single prespecified seed (2024) and the same patient-level fold assignments—yielding the descriptive stability estimates reported in **Table 2**; no selection was performed at this stage. (5) Test evaluation: the locked model was evaluated on the test set exactly once, after the model and evaluation scripts had been frozen. Because the test set was never examined before protocol freezing, no ablation or baseline results led to any design change after test-set evaluation.

**Table 2:** Five-fold stratified cross-validation performance of MSDR-Net on the combined training and validation sets (n = 450).

Fold	Benign/Malignant (n)	Accuracy (%)	Sensitivity (%)	Specificity (%)	Precision (%)	F1 score	AUC
1	70/20	88.9 (80/90)	85.0 (17/20)	90.0 (63/70)	70.8 (17/24)	0.773	0.890
2	70/20	90.0 (81/90)	90.0 (18/20)	90.0 (63/70)	72.0 (18/25)	0.800	0.898
3	71/19	91.1 (82/90)	89.5 (17/19)	91.5 (65/71)	73.9 (17/23)	0.810	0.911
4	70/20	92.2 (83/90)	90.0 (18/20)	92.9 (65/70)	78.3 (18/23)	0.837	0.924
5	70/20	93.3 (84/90)	95.0 (19/20)	92.9 (65/70)	79.2 (19/24)	0.864	0.927
Mean ± SD	—	91.1 ± 1.8	89.9 ± 3.5	91.5 ± 1.4	74.8 ± 3.7	0.817 ± 0.035	0.910 ± 0.016

Each fold contained exactly 90 patients (class distribution differing by at most one case across folds) and was evaluated in a single prespecified run (random seed 2024; Section 2.6). All metrics were computed at the patient level from out-of-fold predictions; integer counts are shown in parentheses. Per-fold confusion matrices are provided in **Supplementary Table S1**.

For the cross-validation stability assessment, each fold consisted of a single prespecified run (random seed 2024). Patient-level out-of-fold predictions were collected for each fold; per-fold metrics and per-fold confusion matrices (**Supplementary Table S1**) were derived from these predictions, overall performance was summarized at the patient level from the pooled out-of-fold predictions, and variability was reported as the standard deviation across folds.

For leave-one-center-out external validation, all data-derived decisions were made exclusively on Institution A data (n = 300): the training/validation partition, five-fold cross-validation, hyperparameter tuning, early stopping, checkpoint selection, and temperature-scaling calibration were all performed within that cohort. The decision threshold (0.5) was prespecified and was not tuned on any data, and preprocessing parameters were fixed, rule-based constants that were never fitted on any study image. The resulting locked model was evaluated once on the independent Institution B cohort (n = 200, including 44 malignant tumors). This cohort was not used in any stage of development of the Institution A model; the model architecture, however, was inherited from the pooled dual-center experiment, in which Institution B had participated. All implementation code, analysis scripts, and trained model weights are publicly available at https://github.com/duan-pytorch/MSDR-Net.

### 2.7 Evaluation metrics and statistical testing

All classification metrics were computed on the independent test set (N = 50) using model outputs at a fixed, prespecified decision threshold of 0.5 on the Softmax-normalized malignant-class probability. Malignant tumors were designated as the positive class and benign tumors as the negative class. Let TP, TN, FP, and FN denote true positives, true negatives, false positives, and false negatives, respectively. The metrics are defined as follows.

Accuracy measures overall classification correctness (Equation 11):

(11) $$\text{Accuracy}=\frac{TP+TN}{TP+TN+FP+FN}$$

Sensitivity (recall) quantifies the malignant tumor detection rate (Equation 12:

(12) $$\text{Sensitivity}=\frac{TP}{TP+FN}$$

Specificity quantifies the benign tumor recognition rate (Equation 13):

(13) $$\text{Specificity}=\frac{TN}{TN+FP}$$

Precision (positive predictive value) measures the reliability of malignant predictions (Equation 14):

(14) $$\text{Precision}=\frac{TP}{TP+FP}$$

The F1 score is the harmonic mean of sensitivity and precision (Equation 15):

(15) $$F_{1}=\frac{2\cdot Precision\cdot Sensitivity}{Precision+Sensitivity}$$

The area under the receiver operating characteristic curve (AUC-ROC) was obtained by integrating sensitivity over (1 − specificity) across thresholds from 0 to 1. AUC was prespecified as the primary performance endpoint, and ConvNeXt-Tiny—the strongest contemporary baseline—was prespecified as the primary comparator; all other pairwise comparisons were considered exploratory.

Rationale for the F1 score: given the 3.5:1 class imbalance (77.8% benign vs. 22.2% malignant), accuracy is vulnerable to inflation by trivial majority-class prediction; an all-benign classifier would achieve ~78% accuracy while missing all malignancies. As the harmonic mean of sensitivity and precision, F1 jointly penalizes missed malignancies and unnecessary referrals, offering a robust summary statistic under imbalance that aligns with the clinical priority of minimizing malignant omissions.

#### 2.7.1 Confidence intervals and statistical testing

Exact 95% confidence intervals for accuracy, sensitivity, specificity, and precision were computed using the Clopper–Pearson method; AUC intervals were estimated using the Hanley–McNeil method. Pairwise AUC comparisons employed the DeLong test for correlated ROC curves, and paired classification discordance was assessed using the two-sided exact McNemar test (R package exact2x2, version 1.6.6). Paired confidence intervals for the differences between MSDR-Net and each comparator in accuracy, sensitivity, specificity, and F1 score were computed with a patient-level paired bootstrap (50,000 joint resamples with replacement; percentile method; random seed 2025), in which patients were resampled jointly and both models’ metrics were recomputed within each resample; paired confidence intervals for AUC differences were derived from the DeLong analysis. All paired intervals are reported in **Supplementary Table S7**. Given the limited test set (N = 50; malignant n = 12), statistical power was anticipated to be insufficient at α = 0.05; exact p-values are therefore reported without Bonferroni correction. With 12 malignant cases, the exact 95% CI for sensitivity spans approximately 38 percentage points (e.g., a point estimate of 91.7% corresponds to 95% CI 61.5%–99.8%); accordingly, this study was designed to estimate model performance with quantifiable precision rather than to demonstrate formal superiority.

#### 2.7.2 Calibration and clinical utility

Calibration was assessed on the independent test set using calibration curves, the Brier score, the calibration slope and intercept, and the expected calibration error (ECE; 10 bins). Because class weighting may distort output probabilities, temperature scaling was additionally fitted on the validation set, and performance metrics are reported both at the prespecified 0.5 threshold and after temperature-scaling calibration (**Supplementary Table S9**). Given the 3.5:1 class imbalance, a precision–recall curve and the area under it (PR-AUC) were also computed. Decision curve analysis was performed to quantify the net benefit of the model against treat-all-as-malignant and treat-all-as-benign strategies across a range of threshold probabilities.

#### 2.7.3 Latency measurement

GPU timing was reported separately for three measures: (1) batch-averaged throughput, measured on a single NVIDIA RTX 4080 at batch size 16 in FP32 precision as the mean of 200 timed runs following 50 warm-up iterations, using CUDA events; (2) single-instance latency, measured with the same procedure at batch size 1; and (3) end-to-end total processing time for the full 50-case test set, including preprocessing. CPU timing was measured separately on an Intel i7-12700K at batch size 1 using wall-clock timing, as the mean of 200 timed runs following 50 warm-up iterations. Computational cost was quantified as multiply–accumulate operations (1 MAC = 1 FLOP) at an input resolution of 224 × 224 using fvcore (v0.1.6), with the identical measurement pipeline applied to MSDR-Net and all comparison models.

#### 2.7.4 Error analysis

In addition to aggregate metrics, a case-level error analysis was conducted by cross-referencing misclassified cases with pathological subtypes and imaging characteristics to identify systematic failure modes.

#### 2.7.5 Comparative experiments and ablation design

Under identical data partitioning and preprocessing conditions, ResNet50 and AlexNet architectures were reproduced and trained using identical strategies (learning rate, optimizer, and number of epochs) to ensure fair comparison. To address the concern that ResNet50 and AlexNet represent classical rather than contemporary architectures, MSDR-Net was additionally benchmarked against EfficientNet-B3 (22), ConvNeXt-Tiny (23), Swin-T (24), and Lead-CNN (25). All comparison models were initialized with ImageNet-1K pretrained weights, with the entire backbone fully unfrozen, and were trained under identical experimental conditions (data partitioning, preprocessing, optimizer, learning rate schedule, and epochs). All efficiency metrics (parameters, FLOPs, and inference latency) were measured under the same hardware and software environment described in Section 2.5.

To verify the contribution of each component, the following ablation variants were configured, forming a complete 2 × 2 factorial design over the two components: (1) MSDR-Net (complete): including MBRB + ECA; (2) Remove ECA (−ECA): removing the channel attention module while retaining MBRB; (3) single-branch convolution without ECA: replacing the three multi-scale pathways in MBRB with a single standard 3 × 3 convolution; and (4) single-branch convolution with ECA retained. All ablation experiments were conducted with identical training hyperparameters and the same run-selection protocol (three seeds, with the run achieving the highest validation AUC carried forward), and each module’s contribution was quantified by comparing test-set performance.

## 3 Results

### 3.1 Patient baseline characteristics and data distribution

This study included 500 pathologically confirmed spinal tumor T2-MR images. Dataset partitioning and demographic characteristics are detailed in **Table 3**, which reports age, sex, center, and pathological subtype distributions separately for the training, validation, and independent test sets, with standardized differences confirming comparability across partitions. Regarding pathological type distribution, among 389 benign tumors (77.8%), hemangiomas were most common (175 cases, 35.0% of the total sample, 45.0% of benign cases), followed by osteochondromas (98 cases, 19.6%) and giant cell tumors (18 cases, 3.6%). Other benign lesions (osteoid osteoma, aneurysmal bone cyst, etc.) totaled 98 cases (19.6%). Among 111 malignant tumors (22.2%), metastatic tumors dominated (86 cases, 17.2%), followed by primary malignancies totaling 25 cases (5.0%): multiple myelomas (17 cases, 3.4%) and chordomas/chondrosarcomas (8 cases, 1.6%).

**Table 3:** Patient baseline characteristics stratified by dataset partition.

Characteristic	Overall (n=500)	Training (n=350)	Validation (n=100)	Test (n=50)	SMD†
Benign, n (%)	389 (77.8)	273 (78.0)	78 (78.0)	38 (76.0)	0.05
Malignant, n (%)	111 (22.2)	77 (22.0)	22 (22.0)	12 (24.0)	0.05
Male sex, n (%)	265 (53.0)	185 (52.9)	53 (53.0)	27 (54.0)	0.02
Female sex, n (%)	235 (47.0)	165 (47.1)	47 (47.0)	23 (46.0)	0.02
Age, mean ± SD	45.4 ± 16.4	45.2 ± 16.5	45.9 ± 16.2	45.7 ± 16.3	0.03
<40 years, n (%)	208 (41.6)	146 (41.7)	41 (41.0)	21 (42.0)	0.01
40–60 years, n (%)	142 (28.4)	99 (28.3)	28 (28.0)	15 (30.0)	0.04
>60 years, n (%)	150 (30.0)	105 (30.0)	31 (31.0)	14 (28.0)	0.04
Institution A, n (%)	300 (60.0)	210 (60.0)	60 (60.0)	30 (60.0)	0.00
Institution B, n (%)	200 (40.0)	140 (40.0)	40 (40.0)	20 (40.0)	0.00
Hemangioma	175 (35.0)	122 (34.9)	36 (36.0)	17 (34.0)	0.02
Osteochondroma	98 (19.6)	68 (19.4)	20 (20.0)	10 (20.0)	0.01
Giant cell tumor	18 (3.6)	13 (3.7)	4 (4.0)	1 (2.0)	0.10
Other benign tumors	98 (19.6)	70 (20.0)	18 (18.0)	10 (20.0)	0.00
Metastatic tumor	86 (17.2)	60 (17.1)	17 (17.0)	9 (18.0)	0.02
Multiple myeloma	17 (3.4)	12 (3.4)	3 (3.0)	2 (4.0)	0.03
Chordoma/chondrosarcoma	8 (1.6)	5 (1.4)	2 (2.0)	1 (2.0)	0.04

Values are n (%) unless otherwise indicated. Pathological subtypes are expressed as counts with percentages of the respective partition. †Standardized mean difference between the test set and the training set; all values were ≤0.10, indicating adequate balance across partitions.

Demographic characteristics showed slightly more male than female patients (265 vs. 235, ratio 1.13:1). Age distribution was highest in the youngest stratum and lowest in the middle stratum, with a slight rebound in the oldest stratum, with an overall mean age of 45.4 ± 16.4 years. The <40 years group comprised 208 cases (41.6%), the 40–60 years group 142 cases (28.4%), and the >60 years group 150 cases (30.0%). Benign tumor patients averaged 39.8 ± 13.2 years, whereas malignant tumor patients averaged 65.1 ± 10.4 years.

### 3.2 Model convergence and cross-validation performance

During training, MSDR-Net converged faster than baseline models, with a steeper initial loss descent and a final training loss of 0.12 versus 0.25 (ResNet50) and 0.45 (AlexNet) (**Figure 3A**). Stepwise learning rate decay at epochs 80 and 140 produced corresponding drops in all loss curves. Validation accuracy (**Figure 3B**) jumped markedly after the first decay, ultimately stabilizing at 93.0% by epoch 200—improvements of 5.0 and 7.0 percentage points over ResNet50 (88.0%) and Lead-CNN (86.0%), respectively, alongside narrower late-phase fluctuations. Five-fold cross-validation on the combined training and validation sets (n = 450), with each fold evaluated in a single prespecified run (Section 2.6), yielded a mean accuracy of 91.1% ± 1.8%, a mean AUC of 0.910 ± 0.016, a mean sensitivity of 89.9% ± 3.5%, a mean specificity of 91.5% ± 1.4%, a mean precision of 74.8% ± 3.7%, and an F1 score of 0.817 ± 0.035 (**Table 2**); per-fold confusion matrices are provided in **Supplementary Table S1**. The maximum fold-to-fold difference in accuracy was 4.4 percentage points.

**Figure 3:**
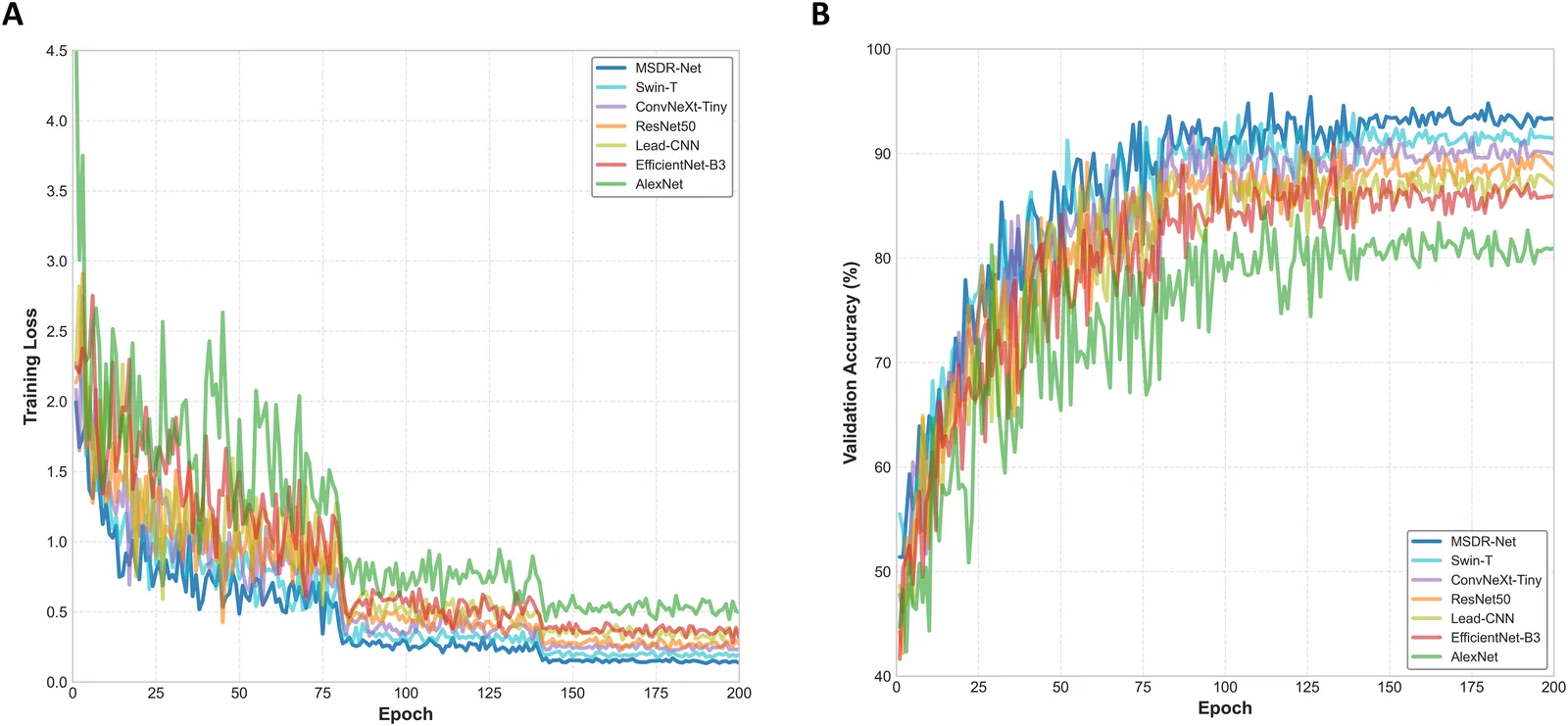
Model training dynamics **(A)** Training loss convergence curve; **(B)** Validation set accuracy curve.

To assess whether the model relied on age-related degenerative changes rather than tumor morphology, we performed age-stratified five-fold cross-validation on the same 450-case cohort. Mean accuracies were 90.1% ± 2.1% (<40 years), 89.3% ± 2.4% (40–60 years), and 92.6% ± 1.9% (>60 years), with corresponding AUCs of 0.898 ± 0.018, 0.891 ± 0.021, and 0.923 ± 0.016. This non-monotonic pattern—in which middle-aged performance was comparable to the youngest group while the oldest group outperformed both—is consistent with the extraction of lesion-related features rather than age-correlated noise; however, stratified performance alone cannot exclude residual age-related confounding, and age remains a potential confounder. This interpretation is further supported by the ablation study: removing the ECA module degraded specificity from 92.1% to 86.8%, implicating background suppression as a major contributor to accurate classification.

### 3.3 Independent test set performance evaluation

On the independent test set (N = 50), MSDR-Net achieved 92.0% accuracy and an AUC of 0.921, the highest point estimates among all evaluated architectures (**Table 4**). ResNet50 and AlexNet achieved 84.0% (42/50) and 78.0% (39/50), respectively; their misclassifications were dominated by false-positive diagnoses of benign mimics—particularly osteochondromas with calcification (ResNet50: 6 FP vs. 2 FN; AlexNet: 9 FP vs. 2 FN)—with occasional false-negative omissions of low-contrast malignant lesions.

**Table 4:** Performance comparison of different models on the independent test set (N = 50).

Model	Accuracy (%)	Sensitivity (%)	Specificity (%)	Precision (%)	F1 score	AUC
MSDR-Net	92.0 [80.8–97.8]	91.7 [61.5–99.8]	92.1 [78.6–98.3]	78.6 [49.2–95.3]	0.846	0.921 [0.812–1.000]
ConvNeXt-Tiny	90.0 [78.2–96.7]	91.7 [61.5–99.8]	89.5 [75.2–97.1]	73.3 [44.9–92.2]	0.815	0.910 [0.793–1.000]
EfficientNet-B3	88.0 [75.7–95.5]	75.0 [42.8–94.5]	92.1 [78.6–98.3]	75.0 [42.8–94.5]	0.750	0.884 [0.758–1.000]
ResNet50	84.0 [70.9–92.8]	83.3 [51.6–97.9]	84.2 [68.7–94.0]	62.5 [35.4–84.8]	0.714	0.838 [0.689–0.987]
Swin-T	88.0 [75.7–95.5]	83.3 [51.6–97.9]	89.5 [75.2–97.1]	71.4 [41.9–91.6]	0.769	0.901 [0.780–1.000]
Lead-CNN	86.0 [73.3–94.2]	83.3 [51.6–97.9]	86.8 [71.9–95.6]	66.7 [38.4–88.2]	0.741	0.886 [0.757–1.000]
AlexNet	78.0 [64.0–88.5]	83.3 [51.6–97.9]	76.3 [59.8–88.6]	52.6 [28.9–75.6]	0.645	0.811 [0.653–0.969]

Values in brackets represent 95% confidence intervals. Accuracy, sensitivity, specificity, and precision were calculated using the Clopper–Pearson exact method; AUC confidence intervals were estimated using the Hanley–McNeil method. AUC was prespecified as the primary performance endpoint, with ConvNeXt-Tiny as the prespecified primary comparator; all other comparisons are exploratory.

ConvNeXt-Tiny and EfficientNet-B3 achieved 90.0% and 88.0% accuracy, with AUCs of 0.910 and 0.884, respectively. Although ConvNeXt-Tiny matched MSDR-Net’s malignant detection rate (91.7%, 11/12), its specificity was lower (89.5%, 34/38 vs. 92.1%, 35/38). EfficientNet-B3’s sensitivity dropped to 75.0% (9/12) despite comparable benign specificity (92.1%). Swin-T achieved a competitive AUC of 0.901 through its shifted-window self-attention mechanism, and Lead-CNN exhibited moderate specificity. Overall, MSDR-Net achieved the highest point estimates in accuracy (92.0%), F1 score (0.846), and AUC (0.921) among all evaluated architectures. Formal DeLong and McNemar tests showed no statistically significant differences between MSDR-Net and ConvNeXt-Tiny (DeLong Z = 0.45, p = 0.653; exact McNemar b = 1, c = 0, p = 1.000) or between MSDR-Net and EfficientNet-B3 (DeLong Z = 1.35, p = 0.177; exact McNemar b = 2, c = 0, p = 0.500) at α = 0.05, reflecting the limited statistical power of the 50-case test set. Complete paired discordance tables for all model-level comparisons are provided in **Supplementary Table S2**, and paired confidence intervals for differences in accuracy, sensitivity, specificity, F1 score, and AUC between MSDR-Net and each comparator are reported in **Supplementary Table S7**. All pairwise comparisons other than the prespecified primary comparison (AUC versus ConvNeXt-Tiny) are considered exploratory. Although formal superiority testing was underpowered, these point-estimate advantages are interpreted as exploratory findings rather than confirmed superiority. A sensitivity analysis excluding the six manually cropped cases in the test set yielded an accuracy of 93.2% (41/44) and an AUC of 0.928 on the remaining 44 cases, consistent with the full test-set results (92.0% and 0.921), indicating that manual cropping had no material impact on the performance estimates.

### 3.4 Error analysis

Confusion matrix analysis (**Figure 4**) revealed that MSDR-Net’s four misclassifications comprised one false-negative malignant lesion and three false-positive benign cases. Subtyping identified osteochondromas with calcification (n = 2) and a vertebral hemangioma with compression deformity (n = 1) as the primary false-positive sources, reflecting known imaging overlap with sclerotic metastases. The single false negative was an early lytic metastatic lesion with poorly defined margins, suggesting that subtle bone destruction without overt cortical breach remains a diagnostic challenge.

**Figure 4:**
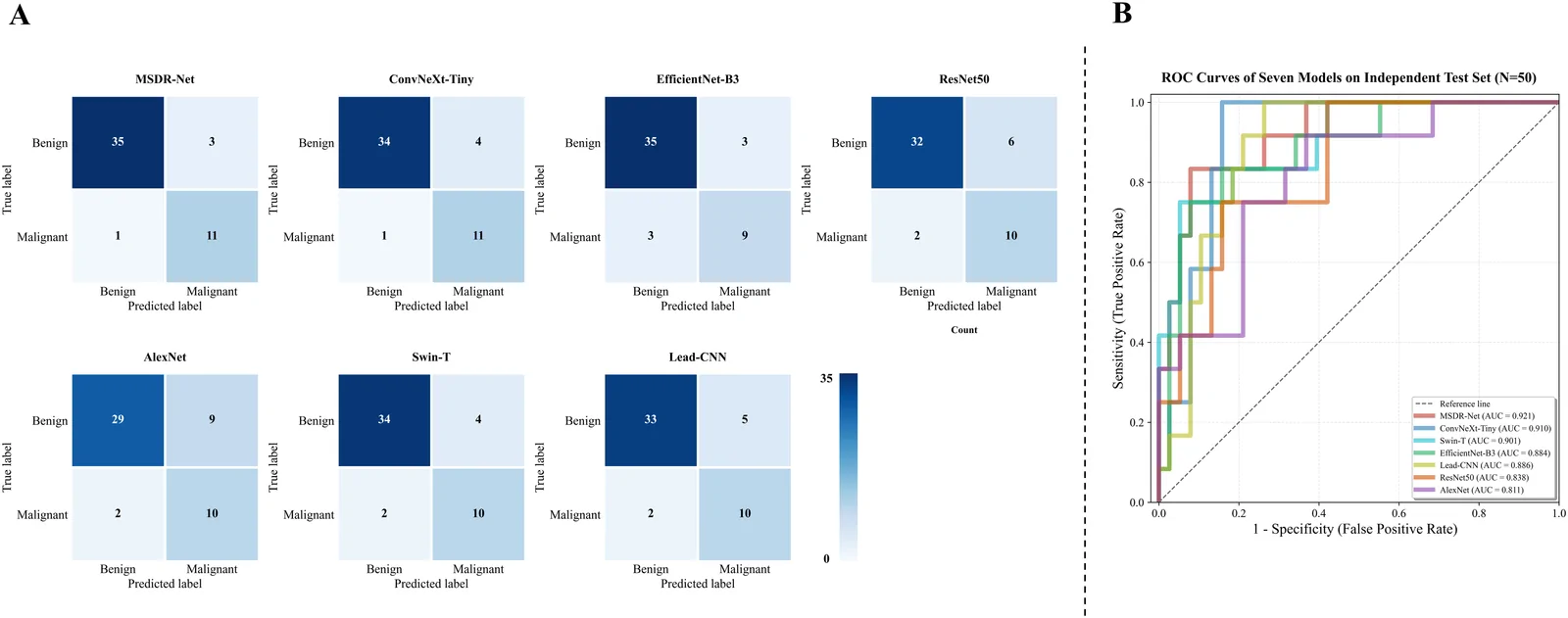
Confusion matrix performance **(A)** and ROC curves of seven models on the independent test set **(B)**.

### 3.5 Center-stratified internal test performance and leave-one-center-out external validation

Center-stratified performance of MSDR-Net on the independent test set is reported in **Table 5**. In addition, we performed leave-one-center-out external validation: the model was developed entirely on data from Institution A (n = 300; training/validation partitioning and five-fold cross-validation were performed within this cohort, Section 2.6) and evaluated once on the independent Institution B cohort (n = 200, including 44 malignant tumors). This cohort was not used in any stage of development of the Institution A model; the model architecture, however, was inherited from the pooled dual-center experiment, in which Institution B had participated. On this external test cohort, the model achieved an accuracy of 86.5% (173/200) and an AUC of 0.875 (**Table 5**; full metrics in **Supplementary Table S3**). Notably, the external cohort contained substantially more malignant cases (n = 44) than the internal test set (n = 12), providing higher precision for estimating malignant detection performance. An additional subtype-stratified analysis of malignant cases in the external cohort (n = 44) showed that detection performance varied by subtype: sensitivity was 88.2% (30/34) for metastatic tumors, 85.7% (6/7) for multiple myeloma, and 66.7% (2/3) for chordomas/chondrosarcomas (**Supplementary Table S10**). These estimates indicate that malignant detection performance was driven primarily by metastatic disease and remains uncertain for rare primary malignancies. Performance on the external cohort was lower than that on the internal test set, underscoring the need for further validation in prospective, geographically distinct cohorts.

**Table 5:** Center-stratified internal test performance and leave-one-center-out external validation performance of MSDR-Net.

Metric	Internal: Institution A (n=30)	Internal: Institution B (n=20)	External: Institution B (n=200)†
Accuracy (%)	93.3 [77.9–99.2]	90.0 [68.3–98.8]	86.5 (173/200) [81.0–90.9]
Sensitivity (%)	85.7 [42.1–99.6]	100.0 [47.8–100.0]	86.4 (38/44) [72.6–94.8]
Specificity (%)	95.7 [78.1–99.9]	86.7 [59.5–98.3]	86.5 (135/156) [80.2–91.5]
Precision (%)	—	—	64.4 (38/59) [50.9–76.4]
F1 Score	—	—	0.738
AUC	0.930 [0.789–1.000]	0.908 [0.720–1.000]	0.875 [0.806–0.944]

Values in brackets represent 95% confidence intervals (Clopper–Pearson for proportions; Hanley–McNeil for AUC). †Leave-one-center-out external validation: the model was developed entirely on Institution A data (n = 300; training/validation partitioning and five-fold cross-validation within that cohort) and evaluated once on the independent Institution B cohort (n = 200, including 44 malignant tumors). This cohort was not used in any stage of development of the Institution A model; the model architecture, however, was inherited from the pooled dual-center experiment, in which Institution B had participated. Full external validation metrics are provided in **Supplementary Table S3**. AUC confidence intervals were truncated at 1.000.

### 3.6 Ablation study: module contribution quantification

To precisely quantify the contributions of multi-scale convolutions and the ECA attention mechanism, this study designed a stepwise ablation experiment (**Table 6**). When the ECA module was removed (retaining the MBRB structure), false-positive misclassifications increased (FP rose from 3 to 5 cases), test set accuracy dropped to 86.0% (43/50), AUC decreased to 0.872, specificity fell to 86.8% (33/38), and sensitivity declined to 83.3% (10/12). This indicates that the ECA mechanism plays a crucial role in suppressing background noise and reducing benign-case misclassification; its absence leads to degraded discriminative ability for complex benign lesions (such as osteochondromas with calcification).

**Table 6:** Ablation study performance decomposition results (independent test set, N = 50).

Model configuration	Accuracy (%)	Sensitivity (%)	Specificity (%)	AUC
Complete MSDR-Net (multi-scale + ECA)	92.0 [80.8–97.8]	91.7 [61.5–99.8]	92.1 [78.6–98.3]	0.921 [0.812–1.000]
Multi-scale, ECA removed	86.0 [73.3–94.2]	83.3 [51.6–97.9]	86.8 [71.9–95.6]	0.872 [0.737–1.000]
Single-path + ECA	84.0 [70.9–92.8]	83.3 [51.6–97.9]	84.2 [68.7–94.0]	0.871 [0.735–1.000]
Single-path, ECA removed	82.0 [68.6–91.4]	75.0 [42.8–94.5]	84.2 [68.7–94.0]	0.814 [0.657–0.971]

Values in brackets represent 95% confidence intervals (Clopper–Pearson for proportions; Hanley–McNeil for AUC). The four configurations form a complete 2 × 2 factorial design over the two components (multi-scale convolutions and ECA). Parameter counts: 19.1M (single-path without ECA), 19.2M (single-path with ECA), and 55.4M (both multi-scale variants; ECA contributes a negligible number of parameters). All variants were trained and evaluated under the identical protocol (three runs with seeds 2021/2022/2023; the run with the highest validation AUC was carried forward to the locked test set).

Furthermore, replacing multi-scale branch convolutions with a single-path standard 3 × 3 convolution (with ECA simultaneously removed) impaired malignant lesion detection capability, with false negatives increasing to three cases, accuracy dropping to 82.0% (41/50), AUC decreasing to 0.814, sensitivity falling to 75.0% (9/12), and specificity declining to 84.2% (32/38). Compared with the complete model, the single-path structure increased the malignant miss rate from 8.3% to 25.0%, highlighting the necessity of multi-scale branch convolutions for capturing subtle malignant signs (such as lytic edges and vertebral destruction).

A third variant retaining ECA on the single-path backbone (19.2M parameters) achieved an accuracy of 84.0% (42/50), a sensitivity of 83.3% (10/12), a specificity of 84.2% (32/38), and an AUC of 0.871 (**Table 6**). Across the resulting 2 × 2 factorial design, ECA improved accuracy by +2.0 percentage points on the single-path backbone and by +6.0 percentage points on the multi-scale backbone, while multi-scale processing improved accuracy by +4.0 percentage points without ECA and by +8.0 percentage points with ECA, supporting a modest super-additive interaction between the two components. Given the 50-case test set and a single locked-test evaluation per configuration, this interaction is presented descriptively and should not be subjected to statistical inference. Because the single-path variants (19.1M–19.2M parameters) are substantially smaller than their multi-scale counterparts (55.4M), these component effects remain partially confounded with model capacity, and the performance deficit of the single-path variants cannot be attributed solely to the removal of multi-scale processing. Neither component alone achieves the full model’s performance.

### 3.7 Computational efficiency, calibration, and clinical utility

MSDR-Net demonstrates a favorable computational profile for potential clinical integration (**Table 7**). The model has 55.4M parameters and a computational cost of 39.8 GFLOPs (input 224 × 224); although this exceeds the budget of ResNet50 (25.5M parameters, 4.1 GFLOPs), it remains well within the capacity of a single consumer-grade GPU while delivering the highest diagnostic accuracy among all evaluated architectures. Although Swin-T is built on the Vision Transformer architecture and features a powerful global self-attention mechanism, its global modeling tends to introduce redundant computations in classification tasks that rely heavily on localized bone-destruction margins (such as spinal tumor T2-MR imaging). Meanwhile, Lead-CNN may loses fine-grained, multi-scale pathological information of subtle lesions through aggressive dimensionality compression, thereby constraining its diagnostic accuracy.

**Table 7:** Computational efficiency and deployment performance comparison of different models.

Model	Parameters (M)	FLOPs (G)	GPU inference (ms)	CPU inference (ms)	Accuracy per million parameters
MSDR-Net	55.4	39.8	12.3 (10.7)	287	1.66
ConvNeXt-Tiny	28.6	4.5	15.3 (13.2)	412	3.15
EfficientNet-B3	12.0	1.80	8.2 (7.1)	195	7.33
ResNet50	25.5	4.1	15.1 (13.2)	350	3.29
Swin-T	28.3	4.48	16.5 (14.2)	440	3.11
Lead-CNN	18.5	2.55	10.8 (9.3)	225	4.65
AlexNet	62.4	0.72	9.8 (8.6)	156	1.25

GPU testing environment is a single NVIDIA RTX 4080; main values report batch-averaged throughput (batch size = 16, FP32), and values in parentheses indicate mixed precision (FP16) mode, measured as the mean of 200 timed runs after 50 warm-up iterations using CUDA events. Single-instance GPU latency (batch size = 1) of MSDR-Net was 18.6 ms; end-to-end processing of the full 50-case test set took 5.1 s (preprocessing ≈ 4.5 s; GPU inference ≈ 0.6 s). CPU latency was measured separately on an Intel i7-12700K at batch size 1 using wall-clock timing (mean of 200 runs after 50 warm-up iterations). Accuracy gain per million params = test set accuracy (%)/number of parameters (M). After INT8 quantization, MSDR-Net inference time is 7.2 ms with 92.0% accuracy on the independent test set (N = 50). FLOPs are reported as MACs at 224 × 224 input, measured with fvcore under an identical convention for all models.

Although EfficientNet-B3 (12.0M, 1.80G) and ConvNeXt-Tiny (28.6M, 4.50G) operate at lower computational budgets, MSDR-Net (55.4M parameters, 39.8 GFLOPs) attained the highest accuracy (92.0%) among all evaluated architectures. By concentrating its representational capacity on multi-scale texture and channel-discriminative features of the lesion, MSDR-Net converts the additional parameters into measurable diagnostic gains, rendering it suitable for integration into clinical decision-support workflows that are highly sensitive to localized pathological findings.

On an NVIDIA RTX 4080 GPU, batch-averaged per-image inference time was 12.3 ms (batch size = 16, FP32; mean of 200 runs after 50 warm-up iterations; Section 2.7), and single-instance latency at batch size 1 was 18.6 ms, with peak VRAM consumption of 2.1 GB; enabling mixed precision (FP16) reduced latency to 10.7 ms and VRAM usage to 1.6 GB. End-to-end processing of the full 50-case test set took 5.1 s (preprocessing approximately 4.5 s; GPU inference approximately 0.6 s). On CPU (Intel i7-12700K, batch size 1, wall-clock timing), per-image latency was 287 ms (approximately 14.4 s for the full test set), remaining below the 500-ms threshold for offline auxiliary diagnosis. Following TensorRT optimization (FP16 + graph optimization), GPU inference time dropped to 8.4 ms; further INT8 quantization (calibration set of 100 validation images) reduced inference time to 7.2 ms, with test set accuracy maintained at 92.0% (46/50), showing no performance degradation compared with the FP32 model.

Parameter efficiency analysis showed that MSDR-Net’s accuracy per million parameters was 1.66 (92.0/55.4), higher than AlexNet (1.25) but lower than Swin-T (3.11), ConvNeXt-Tiny (3.15), ResNet50 (3.29), Lead-CNN (4.65), and EfficientNet-B3 (7.33). The latter two models achieve higher parameter efficiency through aggressive dimensionality reduction; however, this comes at the cost of diagnostic accuracy (86.0% and 88.0%, respectively) and, for EfficientNet-B3, substantially lower malignant detection sensitivity (75.0% vs. 91.7%). This indicates that MSDR-Net achieves a favorable trade-off between accuracy and efficiency, prioritizing clinical detection capability over extreme parameter compression.

Calibration and clinical utility were assessed on the independent test set (**Figure 5A**; **Supplementary Table S9**). The model was well calibrated, with a Brier score of 0.072, a calibration slope of 0.94, a calibration intercept of 0.08, and an expected calibration error of 0.041 (10 bins). Because class weighting may distort output probabilities, temperature scaling was fitted on the validation set; after calibration, the Brier score was 0.068, and classification metrics at the prespecified 0.5 threshold were essentially unchanged (**Supplementary Table S9**). Given the 3.5:1 class imbalance, the precision–recall curve was additionally evaluated, yielding a PR-AUC of 0.82. Decision curve analysis (**Figure 5B**) showed that the model provided greater net benefit than both the treat-all-as-malignant and treat-all-as-benign strategies across threshold probabilities of approximately 0.10–0.60. Derived from a 50-case test cohort containing only 12 malignant tumors, these estimates carry considerable uncertainty and are presented as cohort-specific evidence of technical feasibility; they remain to be confirmed in prospective external validation.

**Figure 5:**
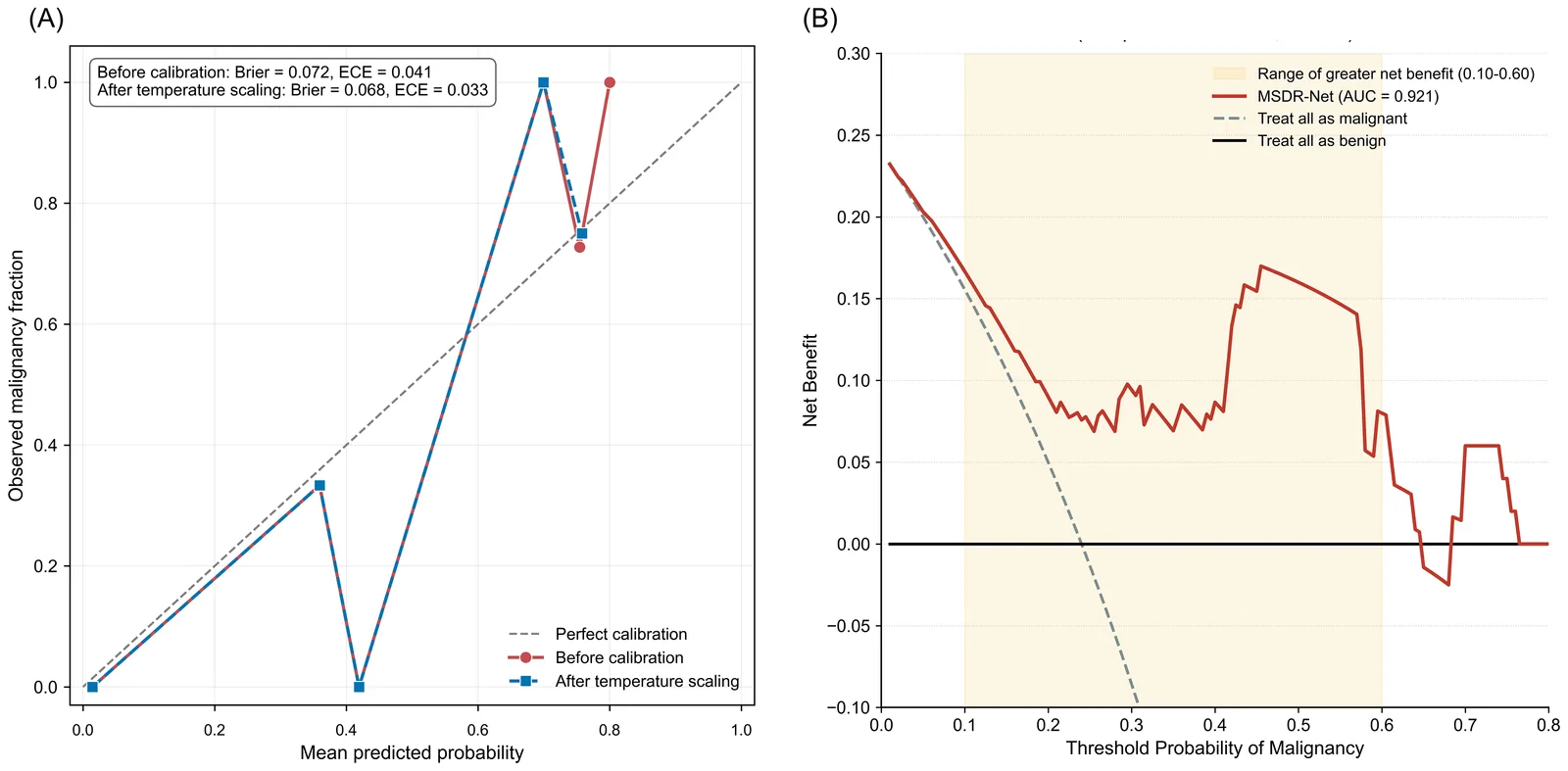
Model calibration and clinical utility of MSDR-Net on the independent test set (N = 50). **(A)** Calibration curve before and after temperature scaling, with the Brier score and expected calibration error annotated. **(B)** Decision curve analysis showing net benefit across threshold probabilities, compared with treat-all-as-malignant and treat-all-as-benign strategies.

### 3.8 Clinical comparison with radiologists

To evaluate the clinical utility of MSDR-Net as an auxiliary diagnostic tool, we performed a blinded reader study against human experts. Three radiologists from the participating centers—a resident (3 years), an attending physician (10 years), and a senior consultant (20 years of spinal imaging experience)—independently interpreted the 50-case independent test set under conditions identical to the model input. Each reader was presented with de-identified ROI-cropped T2-MR images without access to clinical history or pathological results and was required to provide a binary benign/malignant judgment. In addition, each reader rated the likelihood of malignancy on a 5-point confidence scale (1 = definitely benign, 5 = definitely malignant), which was used to construct reader-specific ROC curves and to estimate AUC. Inter-observer agreement among the three physicians was substantial, with pairwise Cohen’s kappa coefficients ranging from 0.71 to 0.76 (mean 0.74), which quantifies the observer variability cited in the Introduction.

As shown in **Table 8**, MSDR-Net achieved an overall accuracy of 92.0% and an AUC of 0.921, numerically higher than the point estimates of all three readers, although the differences were not statistically significant. The model achieved a malignant detection rate of 91.7%, compared with 75.0% for the resident, and its performance closely approximated that of the senior consultant (accuracy 90.0%, AUC 0.908). The error patterns varied markedly by experience: the resident missed three of the 12 malignant lesions—mainly early lytic destruction—while also frequently overcalling benign mimics (five false positives), whereas the attending physician’s errors were concentrated on complex benign subtypes such as osteochondromas with calcification. MSDR-Net showed stable performance across both categories of challenging cases, leveraging its multi-scale convolutions and channel attention to capture subtle malignant signs while suppressing false-positive predictions from benign mimics.

**Table 8:** Performance comparison of physicians and MSDR-Net on the independent test set (N = 50).

Evaluator	Accuracy (%)	Sensitivity (%)	Specificity (%)	AUC
Resident (3 years)	84.0	75.0	86.8	0.852
Attending physician (10 years)	86.0	83.3	86.8	0.884
Senior consultant (20 years)	90.0	91.7	89.5	0.908
MSDR-Net	92.0	91.7	92.1	0.921

Pairwise Cohen’s kappa coefficients among the three readers ranged from 0.71 to 0.76 (mean 0.74). DeLong and exact McNemar tests comparing MSDR-Net with each reader were all non-significant (**Supplementary Table S2**).

Formal pairwise comparisons between MSDR-Net and each reader were conducted using the DeLong test for AUC differences and the two-sided exact McNemar test for paired classification discordance (Section 2.7). Given the limited test set size (N = 50), these tests were underpowered. MSDR-Net did not show statistically significant AUC differences against the senior consultant (DeLong Z = 0.38, p = 0.704), the attending physician (DeLong Z = 0.72, p = 0.472), or the resident (DeLong Z = 0.62, p = 0.535), despite numerically higher point estimates. Exact McNemar tests likewise showed no statistically significant differences in classification agreement between MSDR-Net and any reader (all p > 0.05; complete paired discordance tables in **Supplementary Table S2**). Because no equivalence margin was prespecified and these tests were underpowered at N = 50, the absence of statistically significant differences must not be interpreted as evidence of equivalence between MSDR-Net and the readers. These results indicate that MSDR-Net achieved performance comparable to that of experienced radiologists on this test cohort, rather than definitive statistical superiority, supporting its role as a decision-support tool that may help narrow the diagnostic gap between junior and senior clinicians in resource-limited settings.

## 4 Discussion

To address the persistent clinical challenge of distinguishing benign from malignant spinal tumors on T2-weighted MR imaging, this study introduces MSDR-Net. By synergistically integrating multi-scale feature extraction with an adaptive channel attention mechanism, the model achieved 92.0% classification accuracy and an AUC of 0.921 on the 50-case independent test set of this 500-case dual-center cohort—the highest point estimates among all evaluated architectures. It attained this performance with a modest computational footprint, a property directly relevant to practical deployment. In the blinded reader study, MSDR-Net achieved numerically higher diagnostic accuracy than radiologists at all experience levels, including a senior consultant with 20 years of experience, and a malignant detection rate of 91.7%, compared with 75.0% for the junior resident. Formal statistical superiority was not reached owing to the limited test set size (Sections 3.3 and 3.8); these differences should therefore be interpreted as exploratory advantages in point estimates. Accordingly, we position MSDR-Net not as a replacement for radiologists but as a decision-support tool whose performance was comparable to that of experienced radiologists in this cohort and that may help narrow the diagnostic gap between junior and senior clinicians, particularly in primary care institutions where specialized spinal imaging expertise is scarce.

The architectural design centers on the Multi-Branch Residual Block (MBRB) (26–28). Spinal tumors exhibit marked scale heterogeneity in their MR appearance: microcalcifications and cortical disruptions call for fine-grained capture of local detail, whereas infiltrative growth patterns and vertebral body collapse require macroscopic semantic understanding (29, 30). Conventional single-path convolutional networks, constrained by fixed receptive fields, cannot meet these dual requirements simultaneously. The MBRB overcomes this limitation by parallelizing convolutional pathways with complementary kernel sizes, achieving multi-granularity information fusion with modest parameter overhead. In our ablation experiments, the complete 2 × 2 factorial comparison showed that ECA improved accuracy by +2.0 percentage points on a single-path backbone and by +6.0 percentage points on the multi-scale backbone, whereas multi-scale processing added +4.0 percentage points without ECA and +8.0 percentage points with ECA, indicating a modest super-additive interaction between the two components; because the single-path variants are substantially smaller (19.1M–19.2M vs. 55.4M parameters), these component effects remain partially confounded with model capacity. Unlike encoder-decoder architectures such as U-Net, which rely on complex skip connections and upsampling operations (31, 32), MSDR-Net employs a streamlined five-level encoder that progressively compresses spatial dimensions—a design better aligned with the high-level semantic abstraction required for classification while minimizing computational redundancy. This “divide-and-conquer” strategy for feature extraction mirrors the diagnostic reasoning of experienced radiologists, who integrate local pathological signs with global structural assessment, and may partly account for the model’s performance in this cohort.

When benchmarked against modern efficient architectures, MSDR-Net achieved a higher point estimate of accuracy than ConvNeXt-Tiny (92.0% vs. 90.0%) despite identical malignant detection rates (91.7%), although this difference was not statistically significant (Section 3.3). This exploratory pattern is consistent with the ECA module’s suppression of background noise, which reduces false-positive predictions from calcified benign mimics—a benefit not fully captured by modern training strategies or larger receptive fields alone. The lower sensitivity of EfficientNet-B3 (75.0%) illustrates the risk of aggressive parameter compression in low-contrast medical imaging, where malignant lesions are small and visually ambiguous. These findings support our design rationale: multi-scale convolutions specifically address scale heterogeneity in spinal tumors, whereas compound scaling or generic CNN advances do not guarantee robust malignant detection on T2-MR images.

The incorporation of Efficient Channel Attention further strengthens the model’s discriminative capacity (33–35). In spinal T2-MR images, background anatomical structures (normal vertebrae, soft tissues, and cerebrospinal fluid) often dominate the image, leaving tumor regions relatively sparse and susceptible to noise interference. ECA generates channel-specific weights through one-dimensional convolutional interactions without dimensionality reduction, suppressing irrelevant feature responses while amplifying task-relevant ones. In our experiments, removing ECA alone reduced accuracy by 6.0 percentage points and lowered specificity from 92.1% to 86.8%, highlighting its contribution to reducing false-positive diagnoses. This lightweight attention mechanism introduces minimal computational overhead while improving sensitivity to malignant features, particularly benefiting the detection of low-contrast lesions such as multiple myeloma and early metastatic disease. We caution, however, that these findings are module-level performance observations derived from ablation and do not constitute mechanistic explanations of model decisions.

Although direct cross-dataset comparisons are inherently limited by differences in modality and patient populations, conceptual benchmarking against recent AI literature on bone tumors and spinal imaging helps contextualize the clinical value of MSDR-Net. Wang et al. (36) combined multicenter radiographs with clinical features to develop a deep learning framework for distinguishing primary bone tumors from bone infection; their study enrolled 1,992 patients, and the model achieved AUCs of 0.948 and 0.963 on the internal and external test sets, respectively. Their multimodal fusion and external validation strategies offer useful methodological references for the present study. Xu et al. (37) addressed the scarcity of bone tumor imaging data by employing Pix2Pix and Conv-LSTM-GAN to generate time-series radiographic images, expanding the original dataset approximately tenfold; subsequent 3D-CNN classification of the chemotherapy necrosis rate achieved an AUC of 0.97. Their generative data augmentation strategy offers a practical solution for model training in data-scarce settings. More recently, Natarajan et al. proposed an ROI-guided relational YOLO-SegNet transformer for lightweight joint segmentation and classification of bone tumors on plain radiographs (38), further supporting the feasibility of computationally efficient architectures for skeletal tumor analysis. Radiomics-based machine learning classifiers have likewise shown promise in differentiating malignant from benign bone tumors on plain radiographs, both in unselected cohorts (39) and in specific differentials such as atypical cartilaginous tumor versus high-grade chondrosarcoma (40). For spinal tumor classification, Liu et al. (41) achieved 92.32% classification accuracy for schwannoma and meningioma on MRI using ResNet34-SKConv, validating the discriminative potential of deep learning for primary spinal tumors; however, their approach focused on intradural extramedullary lesions and was limited by a small sample size. Similarly, a weighted-fusion deep learning framework coupling automated lesion detection with multi-network feature integration was developed for benign–malignant diagnosis of spinal tumors on MRI and validated in a cohort of 585 patients (42). By contrast, MSDR-Net attained 92.0% accuracy and an AUC of 0.921 on T2-weighted fat-suppressed MR images—a modality with high soft-tissue contrast for spinal tumor assessment—while requiring only 55.4M parameters and achieving a 7.2 ms INT8-quantized latency, a profile well suited to settings that demand rapid triage of spinal tumors.

The leave-one-center-out analysis provides an initial assessment of cross-institutional generalizability. When the model was developed entirely on data from Institution A and evaluated on the independent Institution B cohort, accuracy decreased from 92.0% on the internal test set to 86.5%, with an AUC of 0.875. This performance decrement reflects both center-related differences in case mix and acquisition protocols and the change in training-cohort size and composition; the two effects cannot be separated in this design, and internal test performance may overestimate real-world performance. At the same time, the external cohort contained substantially more malignant cases (n = 44) than the internal test set (n = 12), allowing malignant detection performance to be estimated with greater precision. Prospective multicenter validation across geographically distinct institutions therefore remains necessary.

From a translational perspective, MSDR-Net demonstrates a favorable deployment profile. Single-image inference requires 12.3 ms on an RTX 4080 GPU and 287 ms on a standard CPU, both well below the 500-ms threshold for offline auxiliary diagnosis. With TensorRT INT8 quantization, latency drops to 7.2 ms without loss of accuracy. Peak VRAM consumption under FP16 is 1.6 GB, and INT8 quantization reduces this footprint further, enabling deployment on the mid-range GPUs or CPU-only workstations common in primary care settings. This balance of accuracy and efficiency places MSDR-Net on a favorable accuracy-efficiency Pareto frontier within this cohort: it achieved higher accuracy point estimates than EfficientNet-B3 and ConvNeXt-Tiny while maintaining lower latency than ConvNeXt-Tiny and ResNet50. Calibration and decision-curve analyses further indicated that the model’s output probabilities were well calibrated (Brier score 0.072; expected calibration error 0.041) and provided net benefit over the treat-all-as-malignant and treat-all-as-benign strategies across threshold probabilities of 0.10–0.60 (Section 3.7). We present these findings as cohort-specific evidence of technical feasibility derived from a small test set: whether the model can be integrated into PACS workflows remains to be prospectively evaluated, and its modest hardware requirements may help mitigate the uneven geographic distribution of specialized spinal imaging expertise, pending prospective validation. We also acknowledge that, although EfficientNet-B3 and ConvNeXt-Tiny are included in the present benchmark, evaluation against additional vision transformers (e.g., DeiT, ViT) and larger Swin variants remains necessary; such large-scale comparative analyses have been incorporated into the protocol of our upcoming multicenter prospective study.

The comparisons with these frontier paradigms also warrant theoretical elaboration. Although Swin-T possesses strong global dependency modeling capabilities, the data-hungry nature of Vision Transformers makes it prone to overfitting on small-sample medical datasets; moreover, the characteristic imaging signs of spinal tumors (such as subtle bone destruction) mostly manifest as localized textural anomalies rather than macroscopic global patterns. Lead-CNN, meanwhile, reduces computational overhead through drastic structural dimensionality reduction, but its excessively compressed routing may discards multi-scale pathological details. In contrast, MSDR-Net combines parallel multi-scale residual pathways with ECA, jointly exploiting multi-scale spatial features and channel-wise recalibration, and thereby achieves balanced screening performance (91.7% sensitivity and 92.1% specificity) at a moderate parameter budget that remains tractable on a single consumer-grade GPU.

Several limitations should be acknowledged. First, the malignant cohort was dominated by metastatic tumors and multiple myeloma, whereas rare primary malignancies comprised only 8 cases; the 92.0% accuracy should therefore not be extrapolated to uncommon subtypes. Calcified osteochondromas and vertebral hemangiomas with compression deformity were the primary sources of false positives. Second, the test cohort was small (N = 50, malignant n = 12), and formal statistical testing was therefore underpowered (Section 2.7); with only 12 malignant cases, the exact 95% CI for sensitivity spans approximately 38 percentage points (point estimate 91.7%, 95% CI 61.5%–99.8%). Accordingly, the study was designed to estimate model performance with quantifiable precision rather than to demonstrate formal superiority, and all test-set estimates should be regarded as preliminary pending confirmation in larger prospective cohorts. Future prospective studies will prespecify a non-inferiority margin, enroll a larger reader sample, and adopt a multi-reader multi-case (MRMC) design to enable formal statistical inference on the comparison between the model and human readers. Third, semi-automated ROI extraction may have introduced selection bias toward lesions with discernible margins; although manually cropped and automatically extracted cases showed no significant differences in demographic or imaging characteristics, such comparisons cannot fully exclude bias related to boundary visibility. Transitioning to automated spine-detection modules and whole-image weakly supervised learning is planned. Fourth, age represents a potential confounder, and single-sequence T2-MR alone may have inherent diagnostic limitations (43–45); future work will incorporate multimodal data. Fifth, although leave-one-center-out external validation was performed, the external cohort originated from a single institution in the same geographic region, and validation at additional, geographically distinct institutions remains future work; our upcoming prospective multicenter study targets at least 50 malignant cases for robust validation. Sixth, no formal interpretability analysis was performed in this study; future work will introduce rigorous attribution methods to characterize the imaging features driving model predictions and will explore whole-image weakly supervised learning paradigms. Seventh, the leave-one-center-out evaluation is not fully independent of Institution B at the design level, because the network architecture and general design choices were finalized in the pooled dual-center experiment, to which Institution B contributed patients; only the data-derived decisions of the external-validation experiment itself were made exclusively on Institution A data.

## 5 Conclusion

MSDR-Net combines parallel multi-scale convolutions with efficient channel attention for the benign–malignant classification of spinal tumors on T2-weighted fat-suppressed MRI. In this 500-case dual-center cohort, it achieved the highest point estimates of accuracy (92.0%) and AUC (0.921) among the evaluated architectures, with a computational footprint compatible with offline clinical use. Performance decreased moderately on the leave-one-center-out external cohort (accuracy 86.5%, AUC 0.875), highlighting the need for prospective validation in geographically distinct populations. Although the model’s point estimates exceeded those of all three readers in the blinded comparison, the absence of a prespecified equivalence margin and the limited statistical power of the 50-case test set preclude any claim of equivalence or superiority over radiologists. Given the small test cohort (12 malignant cases), these performance estimates carry wide confidence intervals and should be regarded as preliminary. Within these limits, the model may serve as an auxiliary decision-support tool, pending prospective validation in larger, geographically distinct cohorts, and its modular design may be adaptable to other skeletal imaging classification tasks.

## Data Availability

The raw data supporting the conclusions of this article will be made available by the authors, without undue reservation.
