# Supplementary material for: Multi-scale convolution with efficient channel attention improves benign–malignant classification of spinal tumors on T2-weighted fat-suppressed MRI
Source: Front Oncol. 2026 Oct 30;16:1951483. doi: 10.3389/fonc.2026.1951483 (PMC13759382; doi:10.3389/fonc.2026.1951483)
Supplement: Supplementary file 1 [file Table1.docx]

**Supplementary Tables**

**Supplementary Table S1. Per-fold confusion matrices of the five-fold cross-validation (patient level).**

| Fold | Benign (n) | Malignant (n) | TP | FN | TN | FP | Correct (n) | Accuracy (%) |
| --- | --- | --- | --- | --- | --- | --- | --- | --- |
| 1 | 70 | 20 | 17 | 3 | 63 | 7 | 80 | 88.9 |
| 2 | 70 | 20 | 18 | 2 | 63 | 7 | 81 | 90.0 |
| 3 | 71 | 19 | 17 | 2 | 65 | 6 | 82 | 91.1 |
| 4 | 70 | 20 | 18 | 2 | 65 | 5 | 83 | 92.2 |
| 5 | 70 | 20 | 19 | 1 | 65 | 5 | 84 | 93.3 |
| Total | 351 | 99 | 89 | 10 | 321 | 30 | 410 | 91.1 (pooled) |

Note: TP, true positive; FN, false negative; TN, true negative; FP, false positive. Malignant tumors were designated as the positive class. Each fold contained exactly 90 patients; each fold was evaluated in a single prespecified run (random seed 2024).

**Supplementary Table S2. Complete paired discordance tables and statistical comparisons between MSDR-Net and all comparators (independent test set, N = 50).**

| Comparison | a (both correct) | b (MSDR-Net only) | c (comparator only) | d (both wrong) | Exact McNemar p | DeLong Z | DeLong p |
| --- | --- | --- | --- | --- | --- | --- | --- |
| MSDR-Net vs. ConvNeXt-Tiny | 45 | 1 | 0 | 4 | 1.000 | 0.45 | 0.653 |
| MSDR-Net vs. EfficientNet-B3 | 44 | 2 | 0 | 4 | 0.500 | 1.35 | 0.177 |
| MSDR-Net vs. ResNet50 | 42 | 4 | 0 | 4 | 0.125 | 1.94 | 0.052 |
| MSDR-Net vs. Swin-T | 44 | 2 | 0 | 4 | 0.500 | 0.82 | 0.412 |
| MSDR-Net vs. Lead-CNN | 43 | 3 | 0 | 4 | 0.250 | 1.29 | 0.198 |
| MSDR-Net vs. AlexNet | 37 | 9 | 2 | 2 | 0.065 | 1.90 | 0.058 |
| MSDR-Net vs. Resident | 40 | 6 | 2 | 2 | 0.289 | 0.62 | 0.535 |
| MSDR-Net vs. Attending physician | 40 | 6 | 3 | 1 | 0.508 | 0.72 | 0.472 |
| MSDR-Net vs. Senior consultant | 44 | 2 | 1 | 3 | 1.000 | 0.38 | 0.704 |
| Senior consultant vs. Resident | 41 | 4 | 1 | 4 | 0.375 | — | — |
| Senior consultant vs. Attending | 41 | 4 | 2 | 3 | 0.688 | — | — |
| Attending vs. Resident | 40 | 3 | 2 | 5 | 1.000 | — | — |

Note: b = cases correctly classified by the first-listed evaluator only; c = cases correctly classified by the second-listed evaluator only. Exact two-sided McNemar p-values were computed with the R package exact2x2 (version 1.6.6). DeLong tests compare correlated ROC curves (AUC). All comparisons were non-significant at α = 0.05 and, except for the prespecified primary comparison (AUC vs. ConvNeXt-Tiny), are exploratory.

**Supplementary Table S3. Leave-one-center-out external validation performance of MSDR-Net (model developed on Institution A, n = 300; evaluated once on Institution B, n = 200).**

| Metric | Value [95% CI] | Counts |
| --- | --- | --- |
| Accuracy (%) | 86.5 [81.0–90.9] | 173/200 |
| Sensitivity (%) | 86.4 [72.6–94.8] | 38/44 |
| Specificity (%) | 86.5 [80.2–91.5] | 135/156 |
| Precision (%) | 64.4 [50.9–76.4] | 38/59 |
| F1 Score | 0.738 | — |
| AUC | 0.875 [0.806–0.944] | — |

Note: The external cohort (Institution B) contained 44 malignant and 156 benign tumors. This cohort was not used in any stage of development of the Institution A model; the model architecture, however, was inherited from the pooled dual-center experiment, in which Institution B had participated. Confidence intervals: Clopper–Pearson (proportions) and Hanley–McNeil (AUC).

**Supplementary Table S4. Inter-reader tumor delineation agreement and validation of the rule-based ROI extraction.**

| Analysis | n | DSC | IoU | HD95 (mm) |
| --- | --- | --- | --- | --- |
| Inter-reader agreement: all cases | 500 | 0.87 ± 0.06 | 0.78 ± 0.08 | 3.4 ± 1.2 |
| Inter-reader agreement: benign | 389 | 0.88 ± 0.05 | 0.79 ± 0.07 | 3.2 ± 1.1 |
| Inter-reader agreement: malignant | 111 | 0.85 ± 0.07 | 0.74 ± 0.09 | 3.9 ± 1.4 |
| Rule-based ROI extraction vs. manual reference | 50 | 0.88 ± 0.05 | 0.79 ± 0.06 | 3.3 ± 1.0 |

Note: DSC, Dice similarity coefficient; IoU, Intersection over Union; HD95, 95th-percentile Hausdorff distance. Values are mean ± SD. Cases with inter-reader DSC < 0.80 were adjudicated by a third senior expert.

**Supplementary Table S5. Layer-by-layer specifications of MSDR-Net (input 224 × 224 × 3).**

| Block | Operation | Kernel / Stride / Padding | Output Size |
| --- | --- | --- | --- |
| Input | — | — | 224×224×3 |
| Stem | Conv + BN + ReLU | 3×3 / 1 / 1 | 224×224×64 |
| Stage 1 | MBRB (1×1 ‖ 3×3 ‖ 5×5) + ECA (k=3) + residual | — / 1 / — | 224×224×64 |
| Down 1 | Conv + BN + ReLU | 3×3 / 2 / 1 | 112×112×128 |
| Stage 2 | MBRB (1×1 ‖ 3×3 ‖ 5×5) + ECA (k=5) + residual | — / 1 / — | 112×112×128 |
| Down 2 | Conv + BN + ReLU | 3×3 / 2 / 1 | 56×56×256 |
| Stage 3 | MBRB (1×1 ‖ 3×3 ‖ 5×5) + ECA (k=5) + residual | — / 1 / — | 56×56×256 |
| Down 3 | Conv + BN + ReLU | 3×3 / 2 / 1 | 28×28×512 |
| Stage 4 | MBRB (1×1 ‖ 3×3 ‖ 5×5) + ECA (k=5) + residual | — / 1 / — | 28×28×512 |
| Down 4 | Conv + BN + ReLU | 3×3 / 2 / 1 | 14×14×1024 |
| Stage 5 | MBRB (1×1 ‖ 3×3 ‖ 5×5) + ECA (k=5) + residual | — / 1 / — | 14×14×1024 |
| Classifier | Global average pooling; MLP (1024→256→2) + dropout (0.5) + Softmax | — | 2 |

Note: MBRB, multi-branch residual block (parallel 1×1, 3×3, and 5×5 convolutions with element-wise fusion); ECA, efficient channel attention with adaptive kernel size k (Eq. 5); BN, batch normalization. Total parameters: 55.4M. The implementation is available at https://github.com/duan-pytorch/MSDR-Net.

**Supplementary Table S6. Hyperparameter search space, selection criteria, and training configuration.**

| Item | Search Space / Setting | Selected / Final Value |
| --- | --- | --- |
| Learning rate (initial) | 10⁻³, 10⁻⁴, 10⁻⁵ | 10⁻⁴ |
| Batch size | 8, 16, 32 | 16 |
| Weight decay | 10⁻², 10⁻³, 10⁻⁴ | 10⁻² |
| Optimizer | AdamW (β₁ = 0.9, β₂ = 0.999) | AdamW |
| LR schedule | MultiStepLR (×0.1 at epochs 80 and 140) | MultiStepLR |
| Epochs / early stopping | 200; patience 20 epochs, monitor validation AUC | 200 / 20 |
| Run and checkpoint selection criterion | Highest validation AUC | — |
| MSDR-Net initialization | Kaiming (random) initialization | — |
| Baseline initialization | ImageNet-1K pretrained, backbone fully unfrozen | — |
| Independent training runs | 3 (random seeds 2021/2022/2023) | — |
| Cross-validation runs | Single prespecified run per fold (seed 2024) | — |
| Class weights | Recomputed within each fold's training portion | w_pos = 2.27, w_neg = 0.64 |
| Data augmentation | Horizontal flip (p = 0.5); rotation ±10°; translation ≤5%; brightness/contrast ±10% | training set only |

Note: The same search space, selection criterion, and training strategy were applied to MSDR-Net and all comparison models.

**Supplementary Table S7. Paired differences (MSDR-Net minus comparator) with 95% confidence intervals on the independent test set (N = 50).**

| Comparator | Δ Accuracy (%) | Δ Sensitivity (%) | Δ Specificity (%) | Δ F1 | Δ AUC |
| --- | --- | --- | --- | --- | --- |
| ConvNeXt-Tiny (primary) | +2.0 [0.0, +6.0] | 0.0 [0.0, 0.0] | +2.6 [0.0, +8.6] | +0.031 [0.000, +0.110] | +0.011 [−0.037, +0.059] |
| EfficientNet-B3 | +4.0 [0.0, +10.0] | +16.7 [0.0, +40.0] | 0.0 [0.0, 0.0] | +0.096 [0.000, +0.267] | +0.037 [−0.017, +0.091] |
| ResNet50 | +8.0 [+2.0, +16.0] | +8.3 [0.0, +28.6] | +7.9 [0.0, +17.5] | +0.132 [+0.027, +0.287] | +0.083 [−0.001, +0.167] |
| Swin-T | +4.0 [0.0, +10.0] | +8.3 [0.0, +27.3] | +2.6 [0.0, +8.6] | +0.077 [0.000, +0.213] | +0.020 [−0.028, +0.068] |
| Lead-CNN | +6.0 [0.0, +14.0] | +8.3 [0.0, +27.3] | +5.3 [0.0, +13.5] | +0.105 [0.000, +0.251] | +0.035 [−0.018, +0.088] |
| AlexNet | +14.0 [+2.0, +26.0] | +8.3 [0.0, +27.3] | +15.8 [0.0, +31.6] | +0.201 [+0.031, +0.398] | +0.110 [−0.004, +0.224] |

Note: Confidence intervals for accuracy, sensitivity, specificity, and F1 score differences are patient-level paired bootstrap percentile intervals (50,000 joint resamples with replacement, with both models' metrics recomputed within each resample; random seed 2025), replacing the Wald intervals reported in the previous version. AUC difference intervals derive from the DeLong analysis and are unchanged. For comparisons with c = 0 (Supplementary Table S2), the paired difference distribution of every threshold-based metric is bounded below by zero; degenerate intervals (0.0 to 0.0) occur when all discordant cases belong to a single class (e.g., sensitivity versus ConvNeXt-Tiny). With few discordant pairs, percentile bootstrap intervals can be anti-conservative; the prespecified exact McNemar and DeLong tests (Supplementary Table S2) remain the primary inferential procedures.

**Supplementary Table S8. Validation-set performance of three independent training runs per architecture (random seeds 2021/2022/2023).**

| Model | Selected configuration (LR / batch / weight decay) | Run seed | Checkpoint epoch (best validation AUC) | Validation AUC | Validation accuracy (%) | Carried forward to test |
| --- | --- | --- | --- | --- | --- | --- |
| MSDR-Net | 1e-4 / 16 / 1e-2 | 2021 | 105 | 0.934 | 92 |  |
| MSDR-Net | 1e-4 / 16 / 1e-2 | 2022 | 127 | 0.941 | 93 | ✓ |
| MSDR-Net | 1e-4 / 16 / 1e-2 | 2023 | 96 | 0.933 | 92 |  |
| ConvNeXt-Tiny | 1e-4 / 16 / 1e-3 | 2021 | 134 | 0.921 | 90 |  |
| ConvNeXt-Tiny | 1e-4 / 16 / 1e-3 | 2022 | 141 | 0.928 | 91 | ✓ |
| ConvNeXt-Tiny | 1e-4 / 16 / 1e-3 | 2023 | 118 | 0.922 | 90 |  |
| EfficientNet-B3 | 1e-4 / 32 / 1e-2 | 2021 | 122 | 0.905 | 88 |  |
| EfficientNet-B3 | 1e-4 / 32 / 1e-2 | 2022 | 135 | 0.912 | 89 | ✓ |
| EfficientNet-B3 | 1e-4 / 32 / 1e-2 | 2023 | 109 | 0.906 | 88 |  |
| ResNet50 | 1e-4 / 16 / 1e-2 | 2021 | 118 | 0.898 | 87 |  |
| ResNet50 | 1e-4 / 16 / 1e-2 | 2022 | 129 | 0.905 | 88 | ✓ |
| ResNet50 | 1e-4 / 16 / 1e-2 | 2023 | 104 | 0.899 | 87 |  |
| Swin-T | 1e-4 / 16 / 1e-3 | 2021 | 126 | 0.914 | 89 | ✓ |
| Swin-T | 1e-4 / 16 / 1e-3 | 2022 | 117 | 0.906 | 88 |  |
| Swin-T | 1e-4 / 16 / 1e-3 | 2023 | 132 | 0.911 | 89 |  |
| Lead-CNN | 1e-3 / 16 / 1e-3 | 2021 | 98 | 0.883 | 85 |  |
| Lead-CNN | 1e-3 / 16 / 1e-3 | 2022 | 112 | 0.891 | 86 | ✓ |
| Lead-CNN | 1e-3 / 16 / 1e-3 | 2023 | 121 | 0.888 | 86 |  |
| AlexNet | 1e-3 / 32 / 1e-3 | 2021 | 92 | 0.845 | 80 |  |
| AlexNet | 1e-3 / 32 / 1e-3 | 2022 | 105 | 0.852 | 81 | ✓ |
| AlexNet | 1e-3 / 32 / 1e-3 | 2023 | 88 | 0.847 | 80 |  |

Note: Checkpoint epoch denotes the epoch at which the best validation AUC was achieved (early stopping monitored validation AUC with a patience of 20 epochs). For each architecture, the run with the highest validation AUC (marked ✓) was carried forward to the single evaluation on the locked test set. Validation accuracy is retained as a secondary column so that previously reported values remain traceable. cuDNN deterministic mode was enabled for all runs.

**Supplementary Table S9. Calibration and clinical utility metrics on the independent test set (N = 50), before and after temperature scaling.**

| Metric | Before Calibration | After Temperature Scaling† |
| --- | --- | --- |
| Brier score | 0.072 | 0.068 |
| Calibration slope | 0.94 | 0.99 |
| Calibration intercept | 0.08 | 0.01 |
| Expected calibration error (10 bins) | 0.041 | 0.033 |
| PR-AUC | 0.82 | — |
| Decision threshold | 0.5 (prespecified) | 0.5 (prespecified) |
| DCA net-benefit threshold range | 0.10–0.60 | — |

Note: †Temperature scaling was fitted on the validation set only. PR-AUC, area under the precision–recall curve; DCA, decision curve analysis (net benefit compared with treat-all-as-malignant and treat-all-as-benign strategies). Classification metrics at the prespecified 0.5 threshold were essentially unchanged after calibration.

**Supplementary Table S10. Subtype-stratified malignant detection performance of MSDR-Net in the leave-one-center-out external validation cohort (Institution B, n = 200).**

| Malignant subtype | n | Correctly detected (n) | Sensitivity (%) |
| --- | --- | --- | --- |
| Metastatic tumor | 34 | 30 | 88.2 |
| Multiple myeloma | 7 | 6 | 85.7 |
| Chordoma/chondrosarcoma | 3 | 2 | 66.7 |
| Overall | 44 | 38 | 86.4 |

Note: Sensitivities are shown with integer counts in parentheses in Section 3.5. The overall row corresponds to the external-cohort sensitivity reported in Table 5.
